# New insights into protein–protein interaction modulators in drug discovery and therapeutic advance

**DOI:** 10.1038/s41392-024-02036-3

**Published:** 2024-12-06

**Authors:** Hossam Nada, Yongseok Choi, Sungdo Kim, Kwon Su Jeong, Nicholas A. Meanwell, Kyeong Lee

**Affiliations:** 1 BK21 FOUR Team and Integrated Research Institute for Drug Development, College of Pharmacy, Dongguk University-Seoul, Goyang, Republic of Korea; 2 Department of Radiology, Molecular Imaging Innovations Institute (MI3), Weill Cornell Medicine, New York, USA; 3 College of Life Sciences and Biotechnology, Korea University, Seoul, Republic of Korea; 4 Baruch S. Blumberg Institute, Doylestown, PA USA; 5 School of Pharmacy, University of Michigan, Ann Arbor, MI USA; 6 Ernest Mario School of Pharmacy, Rutgers University New Brunswick, New Brunswick, NJ USA

**Keywords:** Medicinal chemistry, Drug discovery

## Abstract

Protein-protein interactions (PPIs) are fundamental to cellular signaling and transduction which marks them as attractive therapeutic drug development targets. What were once considered to be undruggable targets have become increasingly feasible due to the progress that has been made over the last two decades and the rapid technological advances. This work explores the influence of technological innovations on PPI research and development. Additionally, the diverse strategies for discovering, modulating, and characterizing PPIs and their corresponding modulators are examined with the aim of presenting a streamlined pipeline for advancing PPI-targeted therapeutics. By showcasing carefully selected case studies in PPI modulator discovery and development, we aim to illustrate the efficacy of various strategies for identifying, optimizing, and overcoming challenges associated with PPI modulator design. The valuable lessons and insights gained from the identification, optimization, and approval of PPI modulators are discussed with the aim of demonstrating that PPI modulators have transitioned beyond early-stage drug discovery and now represent a prime opportunity with significant potential. The selected examples of PPI modulators encompass those developed for cancer, inflammation and immunomodulation, as well as antiviral applications. This perspective aims to establish a foundation for the effective targeting and modulation of PPIs using PPI modulators and pave the way for future drug development.

## Introduction

The study of protein-protein interactions (PPIs) has significantly evolved from early observations of protein complexes in biological systems to a deep and complex understanding of the underlying mechanisms of PPIs.^1–4^ The story of PPIs started with the initial discovery of the first protein structure in 1958 which was followed by rapid technological advancements such as High-throughput screening (HTS) methods have dramatically accelerated the ability to identify PPI modulators.^5–7^ The launch of the Human Protein Atlas project in 2003 marked an important milestone in accelerating the understanding of PPIs research by supplying a comprehensive dataset for protein identification and characterization.^8,9^ The subsequent revolution of cryo-electron microscopy (Cryo-EM) in 2013 further accelerated high-resolution imaging of biomolecules.^10,11^

**Fig. 1:**
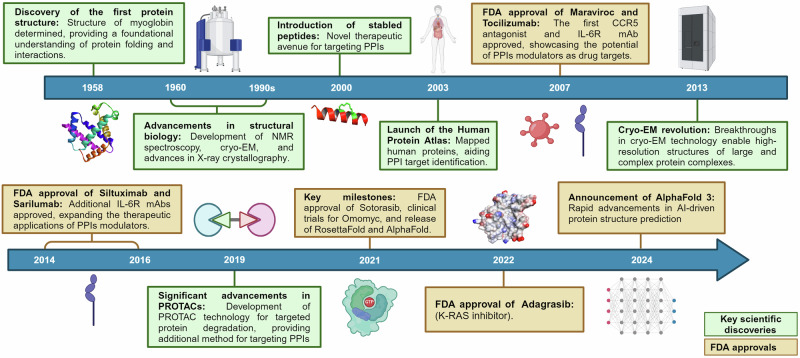
Key milestones in the understanding of PPIs and the development of PPIs modulators. This timeline traces the journey from the discovery of the first protein structure to the emergence of novel research techniques and the translation of fundamental research into therapeutic applications. It highlights pivotal moments in the understanding of protein function and the development of innovative drug modalities discussed throughout this perspective. Created by Biorender

Leveraging these foundational discoveries and incorporating advanced methodologies such as X-ray crystallography, machine learning, and computational capabilities, the development of drug targeting therapeutics for PPIs has made substantial strides. These strides were marked by the FDA approval of PPI modulators such as maraviroc, tocilizumab, siltuximab, venetoclax, sarilumab, satralizumab, sotorasib, and adagrasib for various diseases.^12–17^ Furthermore, rapid advancements in protein structure prediction, exemplified by the simultaneous release of AlphaFold and RosettaFold in 2021, have significantly accelerated PPI therapeutic development.^18,19^ Figure 1 presents a chronological overview of significant advancements and key events in PPI research and therapeutics.

Proteins constitute the fundamental framework for the essential biological processes for all living organisms due to their comprehensive involvement in cell function.^20,21^ These roles include structural support, catalysis, signal transduction, and the transport of molecules amongst many others. However, the crowded cellular environment limits their effectiveness to short-range interactions which is overcome by forming an elaborate network designated as interactomes.^22,23^ These networks allow proteins to communicate and coordinate their activities across the cell, enabling them to perform the complex functions essential for life.^24,25^ The physical interactions between two or more proteins within these networks are designated as PPIs. PPIs occur at specific sites on the surface of proteins described as domain interfaces that can be either transient or stable in nature.^26^ This vital role of proteins necessitates an understanding of their function which is usually elucidated by identifying their binding partners.^27,28^ Understanding the role of at least one interacting component aids in defining its function and pathway within cells, with the mapping of these interactions unveiling the intricate nature of cross-connectivity and cellular pathways. Moreover, this understanding aids in the inference of their dynamic regulation as well as being central to functional genomics and drug discovery.^29^

Many studies have demonstrated that PPIs are primarily influenced by the hydrophobic effect.^30–33^ When this is combined with the fact that PPIs do not share the same structural patterns as enzymes, where the largest or deepest clefts are an indicator of the substrate binding sites, one may conclude that interactions involving enzyme active sites are not typically considered to be PPIs in the context of drug discovery.^34–36^ Instead, binding sites in PPIs usually encompass specific residue combinations, distinct regions, and unique architectural layouts, resulting in cooperative formations referred to as “hot spots.”^37–39^ Hot spots are defined as residues whose substitution, typically by alanine or other amino acids such as glycine and valine with similar disruptive effects, results in a substantial decrease in the binding free energy (ΔΔ*G* ≥ 2 kcal/mol) of a PPI.^40–44^ The energetic contributions of hot spots stems from their localized networked arrangement within tightly packed “hot” regions, enabling flexibility and the capacity to bind to multiple different partners in the intervening spaces.^45^ Such a mechanism explains how a single molecular surface is capable of interacting with multiple structurally distinctive partners whilst also allowing for the targeting of PPIs.

## Advances and challenges in PPI modulator discovery

This section explores the computational landscape for identifying and optimizing PPI modulators and delves into the tools and strategies employed for targeting PPIs. Additionally, the key challenges in small molecule PPI modulation are highlighted along with tactics to harness protein dynamics while achieving optimal selectivity and efficacy in inhibition.

### Strategies in PPI modulator discovery

Rational drug design has demonstrated success in identifying modulators of PPIs by utilizing structural information derived from hot spot analysis.^46^ Additionally, computer modeling techniques coupled with phage display technology has enabled the rational design of peptidomimetics that are designed to recapitulate the secondary structure of key peptide helices, sheets and loops within PPIs. Among the secondary structures employed to design peptidomimetics, the α-helix has been the most widely employed owing to its frequent occurrence and successful targeting.^47–49^ However, the challenging nature of PPI interfaces, which are often flat and featureless, renders traditional rational medicinal chemistry approaches less effective in identifying modulators.^50^

Due to these challenges, multiple approaches have been used to identify modulators of PPIs. High-throughput screening (HTS) is an approach that depends on the utilization of chemically diverse libraries that are often enriched with compounds more likely to target PPIs to successfully identify lead modulators.^51,52^ However, the effectiveness of HTS can be hindered by the lack of specific hot spots on some interfaces which has motivated the application of alternative approaches that are more suitable for the discovery of PPI modulators.^53,54^ Fragment-based drug discovery (FBDD) is one such approach which has been shown to be a useful technique for the design of PPI modulators. The presence of discontinuous hot spots on the surface of many PPI interfaces poses a challenge for HTS but are very amenable to the binding of the smaller, low molecular weight fragments used in FBDD.^55,56^ Interfaces rich in aromatic residues like tyrosine or phenylalanine have been shown to be particularly amenable to fragment hit identification.^44,57^ However, linking these fragments to build a lead molecule is often a challenging task.

The nature of the PPI modulator being developed is another factor that should be carefully considered. For example, PPI stabilizers present a more challenging prospect than PPI inhibitors because, unlike inhibitors that disrupt the interaction interface, stabilizers enhance existing complexes by binding to specific sites on one or both proteins. This necessitates a profound understanding of the intricate forces governing PPI thermodynamics. Stabilizers often act allosterically where their binding site may not be readily apparent in protein structures which hinders the identification of stabilizing moieties.^30,58,59^

The cellular milieu further complicates the development process of PPI stabilizers. Post-translational modifications and other molecules can significantly influence PPI stability.^60^ A stabilizer identified in a controlled in vitro environment might not function effectively within the complex cellular context. The inherent weakness of many PPIs presents another hurdle in the development of PPI stabilizers.^22,61^ Identifying molecules that significantly enhance the stability of these weak interactions necessitates innovative approaches. Traditional HTS methods designed for inhibitor discovery might not be well-suited for identifying stabilizers. In conclusion, developing PPI stabilizers presents a more intricate challenge compared to inhibitors. This is due to the specific binding requirements, the need to consider the cellular context, and the inherent weakness of many PPIs.

### Computational tools for PPI modulator discovery and development

The growing landscape of approved and developing PPI modulators has led to a demand for similar enhancements in computational approaches exploited for the identification and design of these modulators. Traditional computational approaches such as virtual screening have the potential to speed up the discovery process of PPI modulators. Virtual screening can be divided into structure-based and ligand-based approaches.^62,63^ The structure-based approach relies directly on utilizing the structural information of the target protein, while the ligand-based approach screens compounds fitting a pre-built pharmacophore model. Both of these approaches have their merits and limitations; for example, structure-based virtual screening is limited to proteins which have well-known binding pockets, which are often challenging to find in a PPI.^64^ Conversely, ligand-based virtual screening relies upon the exploitation of known potent inhibitors to build a pharmacophore model that can be used for virtual analysis.^65^

However, there are several hurdles which have complicated the deployment of traditional computational approaches toward the identification of novel PPIs and understanding their mechanism of action. Among these hurdles is the dynamic nature of PPIs.^66^ Additionally, the incomplete understanding of the proteome and the gene expression events in an organism’s genome further complicates our understanding of PPIs.^67,68^ Fortunately, the field has witnessed a significant paradigm shift fueled by the rapid progress and widespread adoption of large language models (LLMs) and machine learning (ML) models. This section explores select examples of computational and machine learning applications which have shown potential in accelerating the development of PPI modulators.

## Predicting PPIs

These are a number of computational methods capable of predicting PPIs. Broadly, these computational approaches fall into two categories: homology-based methods and template-free machine learning methods.^69^ Homology-based methods leverage the principle of “guilt by association”.^70,71^ This principle is based on the concept that if a protein shares significant sequence similarity (homology) with a known interactor, it’s likely that these two proteins might interact as well.^72^ Homology-based methods are known for their accuracy and reliability especially in the case of well characterized proteins. However, their applicability is limited when experimentally determined homologs are unavailable.^73^ Template-free machine learning methods are algorithms employed toward the identification of patterns in vast datasets of known interacting and non-interacting protein pairs. These patterns are often represented as features like amino acid sequences, protein structures, or interaction affinities that are used to “train” the ML model. The trained model can then be employed to predict interactions for entirely new protein pairs. Common ML algorithms employed for PPI prediction include Support Vector Machines (SVMs) and Random Forests (RFs).^68,74,75^

In addition to the traditional categorization, the computational methods employed for predicting PPIs can be further categorized based on the type of information utilized. The first category is evolution-based methods which analyze evolutionary relationships between proteins to predict potential interactions. Under the evolution-based method, proteins with similar evolutionary histories are considered to be more likely to interact.^76,77^ Gene-based methods are the second method for predicting PPIs that utilizes gene co-expression data to identify proteins whose genes are often expressed together. In the gene-based method, co-expressed genes often encode proteins that interact functionally.^78–80^ Protein-based methods are a PPI identification methodology that focuses on analyzing the direct physical properties of proteins, such as their amino acid sequences, structures, and predicted binding sites, to predict potential interactions.^80,81^

The synergistic application of these computational tools has the potential to significantly streamline the PPI modulators discovery. Target prediction tools can prioritize targets for further investigation. Once a target is determined, in silico investigations can be carried out to identify the PPI hotspots providing crucial insights into interactions mechanism and providing a target area for modulation. This combined computational approach is becoming more readily available due to the growing body of literature that explores the diverse applications and methods for predicting, analyzing, and storing PPIs.^73,82–85^ Despite the increasing accuracy of these computational tools, rigorous experimental validation remains indispensable to ensure the reliability of predictions and to prevent the pursuit of false leads.

## The identification of PPI hot spots

Identifying hot spots is crucial for the structure-based drug design of PPI modulators. Molecular dynamics (MD) simulations offer a powerful tool toward hot spot identification.^86–89^ MD simulations can sample likely-native conformations and capture the dynamic formation of transient pockets which provide detailed structures for further study. Using MD trajectories, the per-residue binding energies can be calculated using the MM/PB(GB)SA method leading to identifying the energetic contributions of each residue within PPI complex.^90–92^ MD simulations are particularly valuable for studying dynamic features like secondary structures and transient pockets in intrinsically disordered proteins or regions. Furthermore, MD simulations can reveal regions stabilized upon PPI formation by analyzing root-mean-square fluctuations.^93,94^ However, MD simulations are limited by the need for initial 3D complex structures. Currently, the vast number of PPIs far exceeds the available experimental data.

Another key method for hot spot identification is alanine scanning mutagenesis. Alanine scanning mutagenesis (CAS) is a method where functional assays are performed on proteins with specific amino acids mutated to alanine. The advancements in structural bioinformatics have led to the development of in silico CAS. CAS often utilizes MD simulations alongside methods like MM/PBSA calculations to determine the energetic contribution of each residue.^95–98^ The combination of computational methods such as MD simulations, docking and CAS provide a powerful tool for accelerating the identification process of hot spots within PPIs and pave the way for the development of targeted therapeutics through structure-based drug design.

Our previous publication on the gp130-cytokine (IL-6, IL-11, IL-27, OSM) interaction exemplifies the utility of hot spot analysis and molecular docking in elucidating PPIs. The practical application of these computational methods in understanding PPI mechanisms and informing the design of PPI modulators was demonstrated by illustrating the shared IL-6/IL-11 hot spot on the gp130 interface.^99^

## Target identification

One of the challenges in developing PPI modulators lies in the difficulty of identifying suitable targets for candidate molecules. One of the attempts toward accelerating target identification of PPI modulators is PrePPItar, a machine-learning model capable of analyzing PPI targets for drugs.^100^ The core of PrePPItar lies in its ability to integrate diverse data sources which include molecular structures, ATC codes (which denote drug function), side effects, and sequence information for PPIs. By utilizing a machine learning framework with kernel functions, PrePPItar combines these data types into comprehensive similarity profiles for both drugs and PPIs. PrePPItar formulates PPI target prediction as a binary classification problem by leveraging a Support Vector Machine (SVM) model to identify potential drug-PPI associations. PrePPItar demonstrates improved performance when incorporating all data sources compared to methods which only used chemical structure information.^101–103^ By predicting potential PPI targets, PrePPItar expands the search space beyond traditional approaches and can guide future experimental validation making it a valuable tool for advancing research in drug discovery.^100^

## Predicting protein architectures

Despite the widespread availability of protein crystal structures, a significant number of proteins still lack experimentally determined 3D structures due to the challenging nature of X-ray crystallography and cryo-EM modeling for challenging proteins.^104^ However, rapid advancements in computational methods and machine learning have led to the development of tools capable of predicting protein structures from amino acid sequences. Two prominent examples of such tools are Google’s AlphaFold and David Baker’s group RoseTTAFold.^18,19,105–107^ The open-source nature of both tools is poised to significantly accelerate advancements in protein structure prediction and related fields.

### Types of PPI modulators

Current modulators of PPIs can be classified into different categories based on their structural characteristics. The first category is small molecule modulators which are better suited for tight and narrow PPI interfaces. However, these modulators face challenges due to the nature of PPI interfaces which tend to be large, flat, and devoid of the defined pockets that typically characterize small molecule binding sites.^108,109^ This can often necessitate a modulator that covers a substantial surface area and establishes numerous hydrophobic contacts, a profile that frequently introduces pharmacokinetic hurdles due to the large size and poor solubility of such moleules.^50^

Small molecule PPI modulators are classified based on their mechanism of action and site of interaction. For example, small molecules that bind directly to the PPI interface and induce an inhibitory effect are referred to as orthosteric inhibitors^110^ while molecules that bind to a site that is remote from the PPI interface are described as allosteric in function. However, not all modulators of PPIs are inhibitors, with some small molecule modulators stabilizing or even enhancing PPIs. Small molecule modulators acting as PPI stabilizers are commonly referred to as molecular glues and they result in the stabilization of an endogenous PPI or the induction of a non-native interaction. When compared to traditional small molecule PPI inhibitors, molecular glues can offer the advantage of not relying upon high potency or affinity when compared to inhibitors since they do not depend the displacement of a natural binder, but rather they enhance canonical or adventitious interactions. Additionally, molecular glues often bind to a transient and distinctive protein interface, forming a selective interface that minimizes the potential for off-target effects.^111^

Molecular glues can be categorized either based on their binding site or mechanism of action. When classified based on their site of action, molecular glues can act allosterically or bind to the main PPI interface. Allosterically acting molecular glues induce or prevent a conformational change in the target protein which enhances its affinity toward its interacting partner. Meanwhile, molecular glues that bind to a PPI interface exert their stabilizing effect by providing more contact surface area between the two interacting proteins which enhances their binding.^112–114^ When classified based on their mechanism of action, molecular glues are divided into three types. In the first type, molecular glues induce a non-native PPI to “shield” a target protein from performing its normal function. The second type of molecular glue is where the compound inhibits the function of the target protein by redirecting an endogenously formed PPI. Lastly, some molecular glues induce a non-native PPI to generate a novel pharmacological activity.^115^

**Fig. 2:**
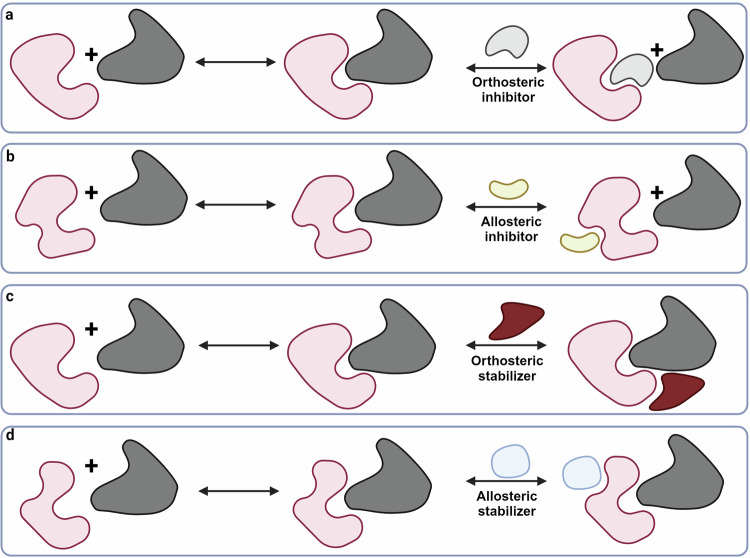
Modes of PPI modulation: orthosteric versus allosteric mechanisms. **a** Orthosteric inhibitors bind directly to the protein-protein interface, competing with one or both proteins for binding. **b** Allosteric inhibitors bind to a distinct site on one of the proteins, inducing conformational changes that disrupt the protein-protein interaction. **c** Orthosteric stabilizers enhance protein-protein interactions by binding to the interface and stabilizing the complex. **d** Allosteric stabilizers bind to a distinct site, promoting a conformation that favors complex formation. Created by Biorender

The design choice of small molecule PPI modulators is largely dependent on the availability of hot spot structural information. In PPIs with known hot spots that converge to establish a possible binding pocket, orthosteric modulators are typically the most prominent. Conversely, allosteric regulation is a more common approach to modulating PPIs where the hot spot structural information is unknown, or a hot spot does not form a suitable binding site. Figure 2 illustrates the binding modes of small molecule PPI modulators.

The second modality with potential for PPI modulation involves the use of inhibitory peptides derived from the binding epitopes of interacting proteins. This approach is based on the fact that specific amino acid sequences mediate PPIs which makes these interactions vulnerable to disruption using peptides based on the sequence under study. Peptides are typically characterized by high target specificity and the ease of adjusting their pharmacokinetics through modifications to their structure.^116–118^ However, there are several limitations that face peptide-based therapeutic development such as the instability of peptides in the cellular environment due to breakdown by enzymes and acidic conditions. Additionally, their larger size compared to small molecule drugs hinders cellular entry and their synthesis can be complex and expensive due to the numerous steps involved.^119–122^

Another limitation of peptide-based therapeutics is the fact that the inhibitory effects of the peptides modulating PPIs depend fundamentally on their structural characteristics both in solution and when bound to the target protein. Typically, isolated peptides are flexible in their unbound state but adopt a well-defined 3D structure upon binding to their protein target. This transition from a flexible to a rigid conformation results in an entropic penalty which can reduce the affinity of the PPI-modulating peptide toward its specific target.^39,123–125^ This issue of an entropic penalty and low cellular uptake can be addressed by modifying the peptides and/or incorporating unnatural amino acids. One such modification is the “stapling” of peptides which restricts the flexibility of a peptide by constraining it into a specific active conformation that pre-organizes it for binding to the target.^126,127^

Several strategies exist for developing therapeutic stapled peptides such as cyclization and ring-closing metathesis. Side chain-to-side chain cyclization has been commonly used for helical peptides and offers the distinct advantage of shielding the peptide backbone from enzymatic cleavage by hindering protease access. Natural peptides and promising candidates from drug discovery often contain disulfide bridges which can be further optimized for stability by replacing one sulfur atom with a carbon atom to establish a cystathionine bridge. Positional scanning is a technique that has been employed to identify optimal locations for these modifications.^128,129^ Meanwhile, ring-closing metathesis utilizes alkenyl side chains to create a hydrocarbon “staple” which has become a popular method for the design of cell-permeable α-helices. This approach offers several advantages including increased efficiency, diverse conformations, and the potential to modify peptides directly during the screening process.^130–132^ β-Turn dipeptide mimetics represent another promising strategy for controlling peptide conformation and mimicking specific secondary structures.^133–135^

The final stages of peptide development involve optimizing solubility and gelling properties for a desired drug formulation. This process is often empirical, relying upon general trends like incorporating hydrophilic and charged residues, minimizing hydrophobic regions, and adjusting the isoelectric point for optimal charge at the formulation pH.^136–138^ Over the past two decades, 28 new peptide-based drugs have been approved globally, with many more in the pipeline. With over 200 peptides in preclinical study and 170 undergoing clinical trials, particularly for metabolic diseases and oncology, the future of peptide therapeutics looks very promising.^139^

Macrocycles are cyclic peptides created by joining the peptide chain into a ring structure and represent another therapeutic option for targeting PPIs.^140,141^ Macrocycles can be either synthetic or naturally occurring where the cyclization imparts a rigid conformation that enhances the stability, protease resistance, and cell permeability of macrocycles.^142,143^ Together these properties marks macrocycles as a highly attractive therapeutic target for developing PPI modulators. Cyclic peptides differ from cross-linked peptides by adopting loop-like conformations. Various strategies, including head-to-tail, head-to-side chain, side chain-to-side chain cyclization and disulfide bond formation have been employed to create both mono- and polycyclic peptides.^144–147^ Although these methods offer diverse approaches to generating macrocycles, only a few approaches have been successfully translated to PPI inhibition.^28^ Overall, macrocycles and stapled peptides share common advantages such as enhanced stability, permeability, and efficacy in targeting PPIs while differing significantly in their structural characteristics and synthetic methodologies.

Another therapeutic option for PPI inhibition is the use of peptidomimetics which are peptide-based molecules that have been tailored from structural insights into aspects of molecular recognition at PPI hotspots and which are capable of maintaining the crucial binding interactions that facilitate expression of high affinity for target proteins.^148–150^ Peptidomimetics can be classified based on their degree of similarity to the natural peptide precursor. In this classification system, there are four classes of peptidomimetics designated A through D.^151,152^ Among these, class A represents the peptides with the highest degree of similarity to their natural peptide precursor, while class D represents the lowest degree of similarity.

Class A peptidomimetics closely resemble the parent peptide and maintain a high proportion of the original amino acid sequence. Class B peptidomimetics involve moderate modifications to the structure of the natural peptide precursor. In class B molecules, the overall structure of the peptidomimetic retains a peptide-like form with some amino acids substituted while backbone modifications are introduced to enhance desired properties. Class C peptidomimetics are highly modified structures with minimal resemblance to the original peptide backbone. Class D peptidomimetics on the other hand are the most distant structurally wise to their natural peptide precursors. Class D peptidomimetics functionally resemble the bioactive peptides but lack the direct link to its side chains.^152–154^ Compared to small molecules, peptides offer heightened target specificity and affinity but can be susceptible to enzyme-mediated degradation if not carefully constructed.^155^

Antibody PPI modulators offer another promising avenue for the development of targeted PPIs therapeutics. However, monoclonal antibodies (mAbs) face several hurdles that limit their use. One major challenge is delivery which is limited by their relatively large size and hydrophilic nature which prevents mAbs from passively crossing cell membranes in the digestive system, rendering oral administration ineffective.^156^ The acidic environment of the stomach leads to potential degradation of mAbs which further restricts oral delivery and necessitate parenteral administration (intravenous, subcutaneous, or intramuscular) for mAbs to reach their targets.^157^ Another limitation of mAbs is their inability to cross the blood-brain barrier (BBB) leading to limited effectiveness in treating central nervous system disorders.^158^ Additionally, mAbs are too large for the standard metabolic pathways located in the kidneys or liver. Instead, mAbs are cleared by the body through two mechanisms: target-mediated clearance (where the mAb-target complex is internalized and degraded by the target cell) and elimination by the reticuloendothelial system which is a network of cells that removes foreign particles from the bloodstream.^159–161^

The two major associated toxicity events are mainly due to target-related effects and/or target-independent toxicities, including immunogenicity.^162^ Target-related toxicities arise from unintended cellular consequences of mAb action which can occur at the intended target tissue or in unintended tissues expressing the target antigen. For example, anti-tumor mAbs targeting the epidermal growth factor receptor (EGFR) can cause skin problems due to the expression of EGFR in skin cells.^163,164^ Meanwhile, immunogenicity refers to the development of anti-drug antibodies (ADAs) by the patient’s immune system in response to the therapeutic mAb. This risk is inherent to all mAbs, regardless of their respective target.^165^ ADAs can lead to a range of complications, including infusion reactions, altered pharmacokinetics properties, decreased target binding and reduced therapeutic efficacy. In severe cases, ADAs might trigger hypersensitivity reactions.^166^ Despite the limitations, toxicity and immunity-related concerns of mAb, they have proven to be successful clinical tools for the treatment of various therapeutic conditions. Over 100 such drugs have already been approved by the FDA, highlighting their efficacy in a range of therapeutic applications.^167,168^

Proteolysis-Targeting Chimeras (PROTACs) are another therapeutic alternative toward PPI modulation. PROTACs consist of an E3 ubiquitin ligase ligand, a protein of interest (POI) ligand, and a linker.^169,170^ The E3 ligand recruits the cellular degradation machinery, while the POI ligand targets the protein for ubiquitination.^171^ In PROTACs the linker design facilitates the formation of a stable ternary complex which brings the POI in proximity to the E3 ligase leading to the promotion of its polyubiquitination and proteasomal degradation.^172^ Unlike traditional small-molecule inhibitors which depend on persistent target occupancy, PROTACs induce ubiquitination through transient binding which leads to target protein degradation and subsequent recycling of the PROTAC.^173–175^ This mechanism offers several advantages over small molecule therapeutics such as the need for lower drug dosage, reduction of potential off-target effects and the ability to overcome resistance mutations that typically render small molecule inhibitors ineffective. PROTACs can also target ‘undruggable’ proteins due to their reliance on minimal binding affinity for the target. Despite these advantages, PROTACs face several limitations such as the limited availability of clinical data on their safety and potential risks which raises concerns about unforeseen side effects and long-term impacts.^176^ Additionally, the dual-targeting nature of PROTACs often results in large molecular weight which hinders the oral bioavailability and tissue penetration.^177^ Furthermore, the complex chemical synthesis and potential for off-target protein degradation are additional barriers toward clinical application of PROTACs.^178,179^ The differences between the different types of PPI modulators are summarized in Table 1.

**Table 1:** The main differences between small molecules, PROTACs, peptidomimetics and monoclonal antibody PPI modulators

Feature	Small molecules	PROTACs	Peptides	Peptidomimetics	Monoclonal antibodies
Size	Low molecular weight	Variable; typically larger than small molecules	Variable; typically smaller than mAbs but larger than small molecules	Variable; typically smaller than mAbs but larger than small molecules	Large proteins
Stability	Generally stable	Potentially less stable than small molecules	Variable; some can be more stable than mAbs	Variable; some can be more stable than mAbs	Less stable; require specific storage conditions
Structure	Simple, well-defined	Bifunctional molecule with ligand and E3 ligase targeting domains	Linear chain of amino acids	Mimics natural peptides	Complex 3D structure
Specificity	Can be non-specific or targeted	Depends on the target protein and E3 ligase specificity	Targeted; specificity depends on sequence	Targeted; specificity depends on design	Highly specific to target antigen
Preferred Route of Administration	Oral, topical, inhalation, Injection	Likely injection due to current limitations	Injection (preferred), potentially other routes depending on design	Variable; can be injectable or potentially oral depending on design	Injection (intravenous, subcutaneous)
Immunogenicity	Generally low	Potential immunogenicity depending on the ligand	Variable; depends on the peptide sequence	Variable; depends on the peptide sequence	Can be immunogenic, leading to reduced efficacy over time
Half-life	Short	Variable; depends on the molecule’s properties	Variable; shorter than mAbs but can be extended with modifications	Variable; can be shorter or longer than mAbs	Long
Metabolism	Metabolized by liver and kidneys	Potentially similar to small molecules	Variable; may require specific clearance pathways	Variable; may require specific clearance pathways	Complex clearance mechanisms
Blood-Brain Barrier Penetration	More facile	Potentially similar to small molecules, limited data available	Variable; depends on the peptide sequence and modifications	Variable; depends on the molecule’s properties	Difficult
Scalability of Manufacturing	Easy and cost-effective	Potentially more complex than small molecules, ongoing research	Variable; depends on peptide sequence and length	Variable; depends on the peptide sequence	Complex and expensive
Drug-Drug Interactions	Higher potential	Potential for interactions depending on the ligand and target protein	Variable; depends on the molecule’s properties	Variable; depends on the molecule’s properties	Lower potential
Advantages	Easy to manufacture, good bioavailability, often low cost	Targeted protein degradation, avoids inducing full protein expression	Can be highly specific, potentially lower immunogenicity than natural peptides	Can target complex structures, potential for oral delivery	High target specificity, potent activity, prolonged time to resistance development, low potential for toxicities
Disadvantages	May have off-target effects, limited target specificity	Still under development, limited clinical data, potential off-target degradation	conformational flexibility, proteolytic instability and poor cellular penetration	Potential immunogenicity, limited stability for some	High cost, complex manufacturing, injection only

### Challenges in small molecule PPI modulation

Extensive scientific study has demonstrated the potential of manipulating PPIs using small molecule modulators as a promising avenue to treating a range of human diseases. These small molecule modulators, which are either natural or synthetic compounds characterized by a relatively low molecular weight, interact with proteins in a way that modifies their function.^50,180^ Moreover, these modulators demonstrate the capacity to selectively bind to specific protein targets with high affinity *via* various mechanisms that includes direct inhibition, allosteric modulation, or stabilization of a PPI.

However, several challenges face the development of small molecule PPI modulators. Among these challenges is the difficulty of identifying lead compounds that effectively target PPIs, especially in cases where naturally-occurring protein-binding small molecules are absent.^181^ Another significant challenge stems from the clustering of “hot” spot amino acid residues situated at the core of protein–protein interfaces, surrounded by less energetically impactful residues that likely shield the surrounding solvent.^182–184^ Additionally, protein–protein interfaces often present flat surfaces (~1000–2000 Å^2^ per side) which lack the kind of defined binding sites (300–500 Å^2^) that can complement small molecules.^46,185–187^ These structural features of PPI binding interfaces have resulted in small molecule PPI modulators exhibiting a larger and more hydrophobic nature when compared to typical orally bioavailable drugs.^109^ Despite these challenges, there has been increasing success in the targeting of PPIs with small molecule modulators, paving the way for extensive drug discovery endeavors.

### Leveraging protein dynamics for targeted therapy

Proteins are not rigid entities since they are constantly undergoing conformational changes which are crucial for their function. The dynamic nature of proteins is particularly evident in PPIs which are critical for signal transduction pathways.^188,189^ The dynamic nature of proteins is a key feature which can be exploited for specific PPI modulation using two key strategies: the targeting of transient states and the exploitation of conformational selection.^190–192^

Many PPIs involved in signal transduction are transient in nature based on fleeting interactions that are mediated by flat interfaces lacking deep cavities. Traditionally, such interactions have been challenging to target due to the requirement for larger and more rigid ligands compared to those used for conventional binding pockets. These ligands are often inspired by natural peptides or proteins and need to account for the flexibility of the solvent-exposed binding site residues. Ideally, the binding site should be able to adopt a preferred conformation for interaction with the regular partner protein while remaining flexible enough to accommodate transient protein states (TPS). Transient protein states refer to the temporary conformations of a protein that exist briefly and are part of the dynamic ensemble of structures that proteins can adopt. These states are often crucial for facilitating interactions with various partners or small molecules, enabling the protein to perform different functions or respond to regulatory signals. The ability to accommodate TPS ensures that the binding site can effectively engage with diverse molecules under varying physiological conditions.^193–195^

**Fig. 3:**
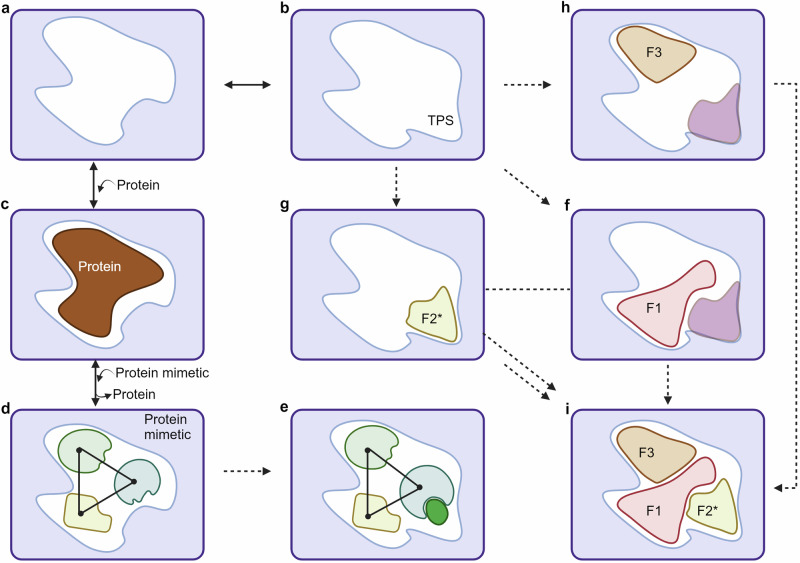
Targeting transient protein states in PPIs for drug discovery. PPI binding sites are flexible, accommodating both favored conformations (**a**) and less preferred transient states (TPS, **b**) that allow for additional interactions. **c** The natural PPI partner recognizes the preferred binding site conformation. **d** Traditional drug discovery aims to mimic natural protein interactions using small molecules, peptidomimetics, or stapled peptides to displace the protein and inhibit the interaction. **e** Knowledge-driven molecular design can target and stabilize transient states using experimental data. However, this approach is impractical without prior knowledge due to the immense task of comprehensively sampling protein conformations. **f** Fragment-based approaches offer a versatile strategy to identify ligands and stabilizers of transient protein states. **f**, **h** Fragments (F1 and F3) can bind to various regions of the binding site, independent of a specific state. **g** Unique fragments (F2) can specifically target and stabilize the transient portion of the binding site (F2*). Fragments identified from transient state binders (F3-F1-F2*) can be linked, extended, or merged (**i**) to create potent inhibitors with high structural complementarity, mimicking classic protein mimetics. Created by Biorender

One approach toward the targeting of transient states involves mimicking the natural protein interaction partner with a small molecule, peptidomimetic, or stapled peptide (Fig. 3). This approach aims to displace the protein and inhibit the interaction but often leads to molecules with high complexity. Moreover, this approach is only viable if the necessary TPS structural information is available. An alternative approach for targeting transient states involves fragment-based screening where small fragments that can bind to various regions of the binding site are identified which potentially includes those specific to the TPS. Linking, growing, or merging these fragments can lead to the development of inhibitors with high structural complementarity capable of mimicking classic protein mimetics but with increased efficacy.^193,196,197^

Traditional drug discovery assumes that small molecules induce a specific conformation in a protein for binding (the induced fit model).^198,199^ However, proteins may pre-exist in a number of conformations, with some being more favorable for binding a specific ligand, and small molecules may preferentially bind to these pre-existing conformations. An understanding of protein dynamics and the pre-existing conformations of proteins can aid in the design of selective modulators with enhanced efficacy.^200–204^ Techniques like molecular dynamics simulations can be employed to provide insight into the conformational landscape of a protein and identify those pre-existing conformations that offer suitability for targeted ligand design.

### Balancing selectivity and efficacy: non-covalent vs. covalent inhibition

The inhibition of biological targets typically involves the attenuation of a protein’s biological function through direct binding which is the result of achieving an equilibrium between the drug and the amino acids of the target protein via multiple non-covalent interactions.^205–207^ These non-covalent interactions include hydrogen bonds, dipole-dipole interactions, van der Waals forces, London dispersion forces and ionic bonds.^208,209^ Non-covalent inhibitors are characterized by their ability to bind to the active site of the target protein with a higher affinity than the target protein’s natural substrate.^210,211^ Together these non-covalent interactions establish a stable drug-protein complex which results in the inhibition of the activity of the target protein. Conversely, covalent inhibition is achieved when ligands containing a reactive functional group (also known as ‘warhead’) such as epoxy, nitrile, or carbonyl group establish a permanent bond with a particular amino acid in the protein, such as serine, cysteine, threonine, or occasionally lysine.^212–214^ This covalent bond formation inactivates the protein for a long time. The warhead group is crucial for this process but can also lead to side effects if it reacts with unintended proteins.^211,215^ Accordingly, the warhead group is essential for this covalent reaction and plays a key role in the potential side effects of covalent inhibitors.

Despite the apparent advantages of covalent inhibitors over their traditional non-covalent counterparts, covalent inhibitors suffer from several limitations. One limitation, is the increased potential for causing side effects due to their indiscriminate irreversible binding.^216,217^ Another challenge is the lack of mainstream computational methods to simulate these irreversible interactions. Such simulations have been proven to be crucial for the understanding and development of non-covalent inhibitors over the years.

## Harnessing the power of PPI modulators

PPIs play key roles in numerous biological processes and their dysregulation, whether through imbalance, overexpression, or under-expression, can lead to various diseases. Modulating these interactions with targeted PPI modulators offers the potential to resolve many clinical conditions driven by such aberrant PPIs. As such PPI modulation is a vast field which would require a book to comprehensively cover all aspects of the topic.^218–223^ This section highlights successful strategies used to develop PPI modulators with significant potential for advancing clinical applications in cancer, inflammatory, and autoimmune disorders, as well as antiviral therapies (Table 2).

**Table 2:** Key examples of clinical applications of PPI modulators

PPI Modulator Target	Target Disease	FDA approved drugs	Mechanism of Action
c-Myc/Max Interaction	Various Cancers	No	
K-RAS PPI inhibitors	Various Cancers	Sotorasib	Inhibits K-RAS G12C mutation by blocking interaction with PDE.
Gp130/IL-6 Interaction	Inflammatory diseases	tocilizumab, siltuximab, sarilumab and satralizumab	Blocks IL-6 signaling by disrupting Gp130/IL-6 interaction.
14-3-3 Protein Interactions	Various Cancers, Neurodegenerative Diseases	No	
HIV-1 gp120 and CCR5 Receptor	HIV	Maraviroc	Blocks CCR5 receptor, preventing HIV-1 from entering host cells.

The PPI modulators featured in this section were carefully chosen to showcase the clinical potential of PPIs, identify modulators with opportunities for further optimization, and offer valuable insights for advancing PPI drug discovery by drawing on successful design strategies as examples. The selection of specific series of small molecule PPIs for SAR analysis was based on the availability of sufficient data to support the prediction of their SAR profiles and their amenability to further optimization in future research endeavors. Ultimately, this section aims to provide insights that can significantly aid future research efforts in PPI modulator development.

### Anticancer PPI modulators

Cancer poses a significant burden on global health with an estimate of 19.3 million individuals received new cancer diagnoses per year. Nearly 10 million of the cancer affected patients die per year emphasizing the urgent need for developing novel cancer therapies.^224^ Various environmental, genetic, and epigenetic factors reprogram cancer-initiating cells which grant them the physical and molecular characteristics needed for tumor growth and therapy resistance.^225,226^ These features, such as sustained proliferation and evasion of growth suppressors, are known as the “hallmarks of cancer” and together they establish a framework linking signaling events to cancer development.^227–229^ PPIs are the fundamental elements within these signaling networks which makes them an ideal target for the development of targeted therapies that can disrupt these crucial interactions.^230,231^

Upon oncogenic stimulation, PPIs play crucial roles in relaying oncogenic signals which facilitate the development of the hallmark cancer features.^232–234^ This process involves everything from receptor engagement with dysregulated growth factors to receptor tyrosine kinase dimerization triggered by gene amplifications or mutations which initiate cascades that promote uncontrolled cell proliferation. For instance, when EGFR is activated due to neurofibromin 1 (NF1) deletion or intrinsic mutations it binds to multiple regulatory proteins which leads to the activation of RAS. This RAS activation then promotes cell proliferation and survival.^235–237^ Meanwhile, cancer progression is promoted by the evasion of growth suppression which is realized by the neutralization of tumor-suppressive functions by PPI complexes such as MDM2–p53 and CDK4–pRB.^225^

Oncogenic network reprogramming results in some PPIs contributing to specific cancer features, while others are essential for multiple cancer characteristics. For instance, the Myc–Max and KRAS/PDE PPIs are involved in evading growth suppression and cell death, as well as promoting genomic instability and altered metabolism.^238^ Consequently, targeting certain critical PPIs may disrupt multiple mechanisms vital for cancer cell survival. Given the extensive involvement of PPIs in driving tumorigenesis through oncogenic network regulation, these PPI interfaces present promising targets for anticancer therapeutic discovery and development. However, challenges remain in developing drugs that specifically target these interactions without disrupting normal cellular functions. Among the various PPIs related to the development and progression of cancer, this section will focus on c-Myc/Max inhibitors and K-RAS/PDE complexes PPI inhibitors.

#### c-Myc/Max inhibitors

**Fig. 4:**
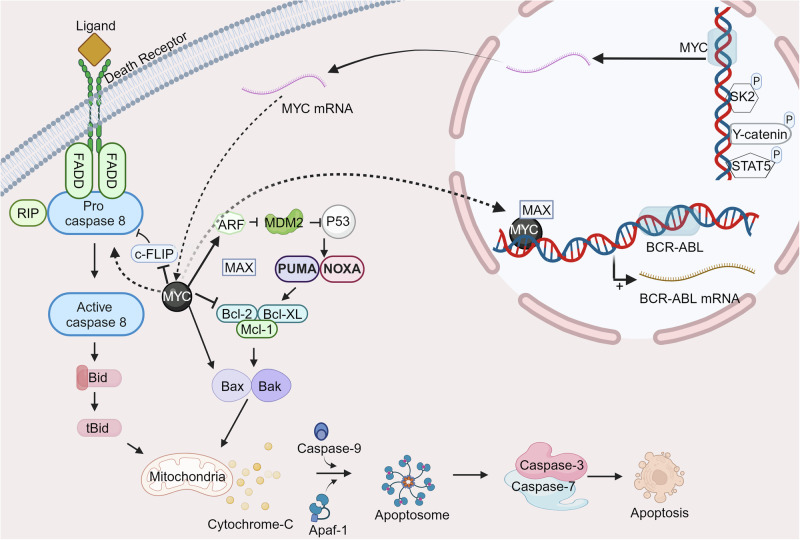
Schematic representation of the c-Myc/Max signaling pathway. The c-Myc oncogene encodes a transcription factor that heterodimerizes with Max to form a complex. This complex regulates the expression of a wide range of genes involved in cell proliferation, growth, apoptosis, and metabolism. Created by Biorender

c-Myc is an oncogenic transcription factor that is characterized by a basic helix-loop-helix leucine zipper (bHLH-ZIP) domain.^239^ c-Myc regulation involves tightly controlled expression and post-transcriptional stabilization mechanisms which are managed via growth-promoting signals.^240^ In genetic model systems, the conditional induction of c-Myc overexpression has been demonstrated to trigger tumorigenesis, while deactivation of the c-Myc-encoding transgene leads to sustained tumor regression.^241,242^ The biological activity of c-Myc is intrinsically dependent on the formation of a heterodimer with its partner protein Max.^243^ Unlike the monomeric form of c-Myc, the c-Myc/Max heterodimer adopts a structured coiled-coil configuration possessing ~70% α-helical content, which escalates to 84% upon binding to DNA.^244^ The signaling pathway of c-Myc/Max is illustrated in Fig. 4.

The c-Myc/Max interaction can be disrupted by two main therapeutic approaches of which the first involves inhibiting the c-Myc/Max PPI while the second exploits the stabilization of Max homodimers thereby limiting the availability of Max for association with c-Myc.^245,246^ The first approach directly targets the PPI between c-Myc and Max using small molecules which are designed to bind to the interface where c-Myc and Max interact which prevents the formation of the c-Myc/Max complex.^247,248^ The second strategy takes a more indirect approach by stabilizing Max homodimers with the aim of promoting the formation of Max homodimers to limit the availability of free Max.^249–251^ However, the approach of stabilizing Max homodimers is associated with unintended consequences on other Max functions. Evidence has been gathered to suggest that inhibiting Myc markedly impedes tumor progression and cell survival regardless of its normal or dysregulated state in tumors.^246,252,253^ Moreover, despite the fact that c-Myc is widely expressed in normal proliferating cells, in vivo studies have demonstrated that long-term and whole-body genetic silencing of c-Myc resulted in remarkably mild and reversible side effects.^254–256^ Together, these findings indicate that the pursuit of modulators of the c-Myc/Max interaction offer a viable and promising anticancer therapeutic target.

However, attempts to target c-Myc have met with considerable difficulty due to the intrinsically disordered nature of the bHLH-ZIP domain.^257,258^ Nevertheless, in spite of this challenge, there are several successful examples of targeting the c-Myc/Max interaction that can be classified based on their mechanism of action.

##### c-Myc/Max small molecule inhibitors

**Fig. 5:**
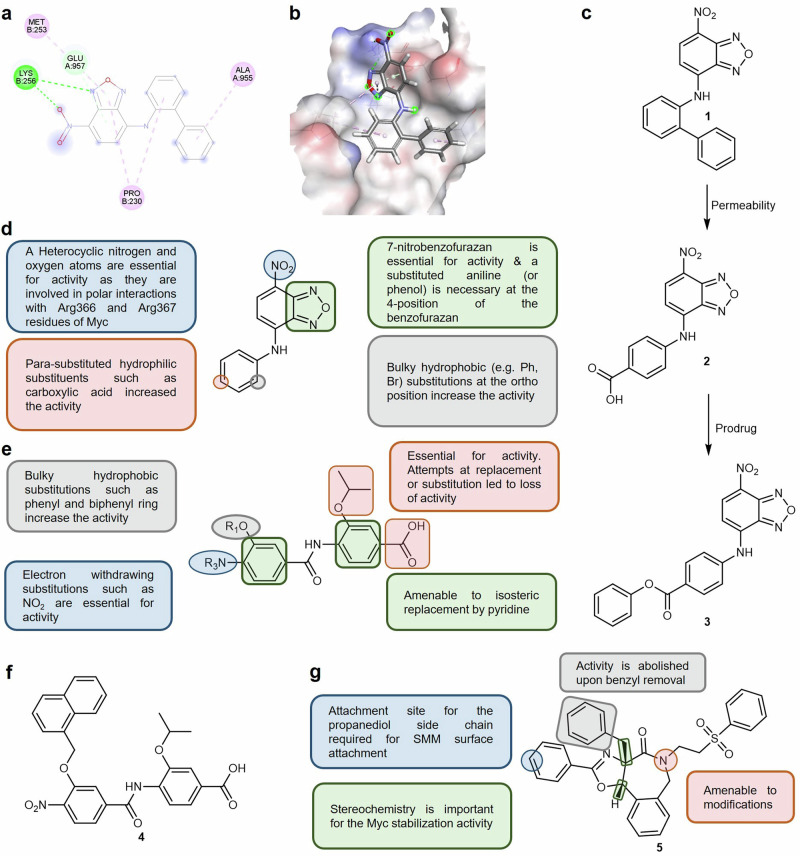
SARs and mechanism of action exhibited by the direct c-Myc inhibitor 1 and c-Myc/Max α-Helix small molecule modulators. 2D (**a**) and 3D (**b**) representations of the binding interaction between compound 1 and c-Myc, based on identified hot spots (PDB: 1NKP).^474,475^ **c** Chemical structures of **1**, its carboxylic acid derivative **2**, and potential prodrug **3**. **d** SAR associated with **1** and its derivatives. **e** SARs associated with c-Myc/Max heterodimer α-helix mimetics. **f** Chemical structure of the α-helix mimetic **4**. **g** SARs associated with the direct c-Myc small molecule stabilizers **5**

*Direct c-Myc small molecule Inhibitors:* The main approach that has been used to modulate the c-Myc/Max interaction has been the direct targeting of one of the three distinct binding sites present in the 85-residue bHLH-ZIP domain of the c-Myc transcription factor.^258,259^ These three binding sites are present in the region defined by residues 363-381 which are located at the junction between the DNA-binding domain and helix 1. When small molecule inhibitors bind to these sites, localized conformational alterations are introduced that maintain the general disorder of c-Myc while preventing its dimerization with Max. Among the direct c-Myc Inhibitors, 10074-G5 (**1**) (Fig. 5) represents a promising lead for further optimization for several reasons. The first is its exceptional feasibility due to the simplicity of synthetic access which relies upon a single-step preparation from commercially available materials. Secondly, the modular nature of its structure, which is based on three distinct embedded moieties, make it amenable to facile structural variation.

Based on the promising activity displayed by **1** and its derivatives, the derived SARs (Fig. 5) indicate that the 7-nitro group and the 1,2,5-oxadiazole moiety in the benzofurazan ring are essential for c-Myc modulation. The significance of the nitro group was attributed to polar interactions established between the heterocyclic nitrogen and oxygen atoms and the binding residues of c-Myc (Fig. 5a). This was further confirmed when the nitro substituent was replaced with an N-acyl carboxylic acid derivative which exhibited an increased inhibition of Myc–Max heterodimer formation. Meanwhile, the benzofurazan ring was found to be tolerant of *ortho*- or *para*-substitution at position 4. *Ortho*-substituents on the benzofuran ring necessitated a bulky hydrophobic group such as a phenyl ring or a bromine atom. On the other hand, a hydrophilic moiety such as a carboxylic acid was preferred as a *para*-substituent of the benzofurazan ring, a molecular edit that resulted in a significant increase in both inhibitory activity and solubility. Regrettably, the improvement in activity observed with the introduction of the *para*-disposed carboxylic acid substituent was accompanied by lowered cell permeability which was attributed to the polar nature of this moiety which resulted in poor in vivo activity.^259–261^

In spite of its promising activity profile, the poor solubility and poor metabolic stability of **1** indicate that further modifications are required.^262,263^ Given the predominantly aromatic nature of **1** and its derivatives, one approach to improve its solubility would involve modulating the degree of unsaturation. The rational and gradual reduction of double bonds, informed by both rational design and experimental data, is a proven strategy capable of maintaining binding affinity while enhancing solubility.^264,265^ This strategy is based on the principle that increased unsaturation generally correlates with lower water solubility, as highlighted by the lower average ring count in successful oral drugs.^266,267^ Therefore, targeted modification of the unsaturation profile within **1** presents a promising avenue for future modification.

The design and evaluation of prodrugs of **1** is another potential avenue that could enhance the pharmacokinetic and pharmacodynamic properties of the molecule. One such strategy was attempted where the *para*-carboxylic acid of **2** was esterified to afford prodrug **3** (Fig. 5c). Esterification successfully enhanced cell permeability which led to efficient intracellular accumulation of **2** following application of **3** However, **3** displayed susceptibility to extracellular esterases which resulted in depletion of the extracellular reservoir. Additionally, while the cells retained **2** for extended periods, a significant portion became localized within the cytoplasm which reduced the Myc inhibitory activity. These findings highlight the need for developing prodrugs of **1** which can maintain persistently high extracellular levels while exhibiting minimal susceptibility to extracellular degradation.

*c-Myc/Max heterodimer small molecule inhibitors:* The second approach employed to develop c-Myc/Max small molecule inhibitors involves the use of α-helix mimetics aimed at the disruption of the coiled-coil structure that mediates the c-Myc/Max heterodimer interaction. This approach prevents the heterodimer from binding to DNA without inducing the dissociation into the monomeric c-Myc and Max components.^268^ This strategy holds substantial promise since it overcomes the inability of direct c-Myc inhibitors to disrupt established dimers which are characterized as possessing a high free energy of protein–protein association.^239,258,269^ This hypothesis was validated by a novel series of biphenyl-based α-helix mimetics which demonstrated the ability to bind to the helical form of c-Myc. This binding resulted in the disruption of the c-Myc/Max heterodimer’s capacity to bind to DNA while not causing the dissociation of c-Myc/Max heterodimers.^268^

The designed mimetics feature a hydrophobic core flanked by electron-rich peripheries which were specifically designed to target the hydrophobic domain of helical c-Myc responsible for the formation of the rigid tertiary structure that is formed upon dimerization with Max. The intended disruption of the c-Myc/Max dimer was established employing several validation techniques that included NMR spectroscopy, heteronuclear single quantum coherence spectroscopy (HSQC) and surface plasmon resonance (SPR). The SARs associated with the synthetic α-helix mimetics are summarized in Fig. 5e and show that bulky hydrophobic substituents such as phenyl and biphenyl rings at the R_1_ position significantly enhanced inhibitory activity. Electron-withdrawing substituents such as NO_2_ at the R_3_ position are essential for inhibitory activity while both phenyl rings were found to be amenable to isosteric replacement by pyridine. The presence of the isopropyl and carboxylic acid moieties on the benzoic acid ring were identified as crucial for the expression of inhibitory activity. This was highlighted by significant decreases in c-Myc/Max inhibitory activity when the acid was converted to an ester or an amide or the isopropyl group was replaced with larger aliphatic or aromatic groups. This loss in activity suggests that these groups play key roles in the binding interaction or the structural configuration contributing to the inhibitor’s binding shape.

Among the synthesized compounds, **4** (Fig. 5f) was the most potent with a binding affinity (*K*_d_) of 10 μM. Moreover, **4** was able to induce cell cycle arrest and inhibit c-Myc-dependent gene expression. However, off-target activity as well as non-specific toxicity were observed when **4** was evaluated in more detail, indicative of the need for further optimization. One of the strategies that has been successfully employed to improve off-target activity is the exploitation of the structural features of the binding domain to aid in the modification of derivatives. This strategy proved to be highly successful in enhancing the selectivity of 14-3-3 molecular glues, a detailed discussion of which is presented later in the discussion.

Meanwhile, one of the main factors associated with toxicity in small molecules is the presence of structural fragments, commonly referred to as toxicophores, that can be associated with adverse outcomes.^270,271^ One such a toxicophore is the nitro group that is present in **4** and which could be a contributing factor to the observed toxicity due to the reported metabolic activation of the NO_2_ moiety to a nitrenium species that is a known mutagen. Accordingly, the first step in resolving the observed toxicity of the c-Myc/Max mimetics would be to investigate the effect of the different derivatives on the observed toxicity in a toxicity study. If the NO_2_ group is proven to be the main contributing factor toward the toxicity, there are two rational strategies have been reported to mitigate this toxicity. The first would be to replace the nitro group with other electron withdrawing moieties and testing their activity. Alternatively, the metabolic activation of NO_2_ can be mitigated by introducing bulky substituents such as alkyl substituents near the nitro group, thereby creating steric hindrance that can interfere with metabolic activation.^272,273^ Given that bulky substitutions have been observed to increase the activity and that the NO_2_ is essential for activity (Fig. 5), the latter approach could be more effective for identifying more effective c-Myc/Max mimetics.

*Direct c-Myc small molecule stabilizers:* KI-MS2-008 (**5**) is an asymmetric polycyclic lactam identified through screening of unbiased small molecule microarrays which represents a groundbreaking approach to combating Myc-driven cancers.^249^ KI-MS2-008 (**5**) directly binds to Max and stabilizes homodimer formation (IC_50_ = 2.15 μM after 3 days) which lead to mimicry of the effect of Myc loss. Notably, **5** effectively reduces Myc protein levels which disrupts Myc-dependent transcription and suppresses tumor growth in both cellular and murine cancer models including T-ALL and HCC. Examining **5** and its derivatives has led to the identification of several SARs (Fig. 5g). The azepane ring was amenable to modifications, maintaining activity upon ring opening or different substitutions at the nitrogen site. Removal of the benzyl substituent abolished Max stabilization activity, indicating its essentiality. Additionally, the stereochemistry of the core substitutions greatly affected activity, while the propanediol side chain, predicted to mediate attachment to the SMM surface, was not required for activity. These findings offer compelling evidence for targeting Max as a viable cancer therapy strategy and mark **5** as a valuable tool for the development of improved therapeutics and further exploration of Max as a promising drug target.^249^

##### c-Myc/Max protein-based inhibitors

The intrinsically disordered nature of MYC means that it is undergoing constant changes which complicates the design of small molecule inhibitors due to the lack of a stable binding pocket.^274,275^ The disordered nature of MYC has directed effort toward finding alternate therapeutic options for MYC inhibition. One such solution is Omomyc, a specially designed mini protein derived from MYC itself.^276^ Omomyc directly binds to MYC which disrupts the heterodimerization of MYC/MAX. Omomyc mini proteins have demonstrated the unique ability of targeting all three forms of MYC which prevents the activation of genes typically controlled by MYC.^277–279^

Omomyc was initially used as a MYC inhibitor within cells and later demonstrated efficacy against transformed cells while minimally affecting normal cell proliferation.^279–281^ Interestingly, the theorized application of Omomyc was originally deemed implausible due to the difficulty of achieving a deliverable expression of Omomyc peptides and lack of translation to in vivo models.^278,282^ Subsequent testing in mouse models revealed Omomyc to possess efficacy and a remarkable therapeutic window across various tumor types, regardless of their origin or driving mutations. These findings caused a shift in the perception of MYC as a druggable target which up to that point was still considered as proof of concept.

The subsequent discovery of the unexpected cell-penetrating properties of Omomyc shifted the view of Omomyc and its potential as a viable drug candidate. Over two decades after its initial discovery, Omomyc, which is now known as OMO-103, entered phase I clinical trials in 2021 where it demonstrated promising safety and clinical activity in patients with various solid tumors.^283^ These results have paved the way for a new trial investigating OMO-103 in combination with chemotherapy for pancreatic cancer. Omomyc’s journey underscores the challenges and successes of targeting proteins once deemed “undruggable.” The advancement of OMO-103 into clinical trials brings significant hope for MYC inhibition in oncology and the use of mini proteins as a viable therapeutic option.

#### K-RAS PPI inhibitors

**Fig. 6:**
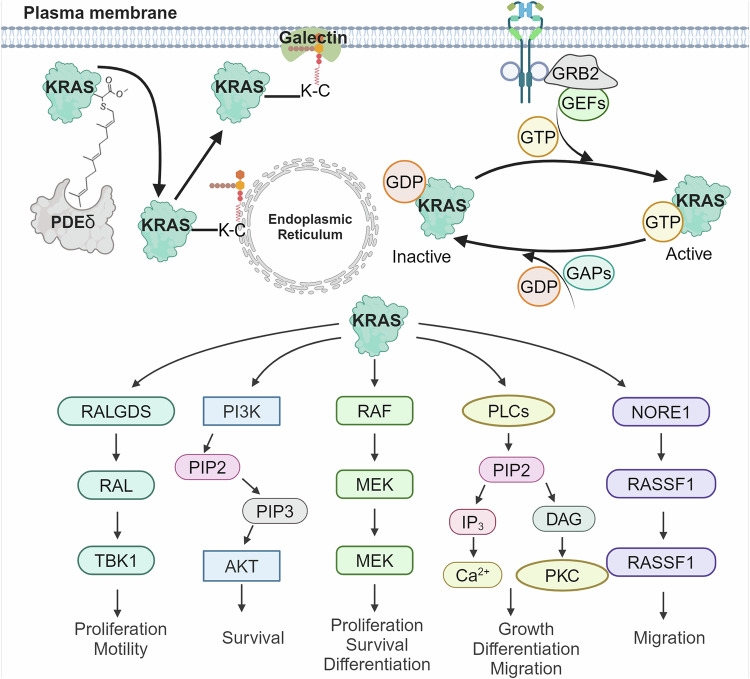
Schematic representation of the signaling cascade of K-RAS. Upon activation by upstream growth factor receptors, K-RAS undergoes conformational changes, leading to the recruitment and activation of downstream effector proteins, including RAF and PI3K. These proteins initiate complex signaling cascades that regulate cell proliferation, survival, differentiation, and metabolism. Key downstream effectors and their biological functions are highlighted. Created by Biorender

The RAS family, composed of H-, K-, and N-RAS, are oncoproteins that have been heavily linked with cancer development and tumor promotion, with RAS mutations occurring in about 20–30% of human cancers.^284,285^ The RAS family acts as membrane-bound molecular switches when activated by guanine nucleotide exchange factors (GEFs) that promote the change from the inactive “GDP-bound” state to an active “GTP-bound” state.^284,286^ The activated GTP-RAS initiates a downstream signaling pathway that, in turn, leads to the activation of various effectors including PDEδ, PI3K, RAF, AFAD, TIAM1, and IMPA1 among many others (Fig. 6).^287,288^ While RAS oncoproteins share a similar overall structure, they are distinguished by their hypervariable regions (HVR). The HVR domain acts as a fingerprint that influences the behavior of the membrane-bound RAS and the way it interacts with its surroundings. These distinct interaction sites allow for the complex regulatory functions of KRAS, where it can interact with multiple proteins simultaneously to control cellular processes.

H-RAS and N-RAS rely primarily on additional “palmitoylation” modifications which mediate membrane tethering but which is absent in K-RAS. Thus, K-RAS relies on an interaction with the phosphodiesterase 6 delta subunit (PDEδ) that facilitates the proper processing of the farnesylated and methylated cysteine at the C-terminus of its hypervariable region. This interaction promotes K-RAS solubilization and subsequent targeting to the endoplasmic reticulum (ER). Subsequently, PDEδ transports K-RAS to the perinuclear membranes for plasma membrane re-localization in a process facilitated by the ADP ribosylation factor-like GTPase 2 (Arl2). Notably, while PDEδ interacts with H- and N-RAS, that interaction is reliant on a Golgi-based de-palmitoylation/re-palmitoylation cycle for plasma membrane enrichment.^289–293^

Mutations in the RAS oncogenes, particularly K-RAS, have been found in approximately 30% of human tumors.^294^ Moreover, almost all pancreatic ductal adenocarcinomas exhibit dependence on mutant K-RAS. Together, these observations highlight K-RAS as a key therapeutic target for anticancer drug development.^295,296^ The development of K-RAS-targeted therapeutics has been hindered by its relatively flat surfaces and picomolar affinity for nucleotides.^297,298^ However, the recent approval of MG510 (sotorasib) and MRTX840 (adagrasib) highlight the druggability potential of the G12C form of K-RAS.^16,17^

##### K-RAS small molecule inhibitors

This section presents examples of specific K-RAS small molecule inhibitors by focusing on their binding mechanisms and SAR. This approach is intended to provide insights into successful development strategies and accelerate future advancements, without delving into the effects of their inhibition on specific K-RAS partners. While the approaches described above yielded potent K-RAS inhibitors, their potential clinical application is currently limited due to their reported non-specific cytotoxicity or low cellular uptake. An alternate pathway to target the K-RAS PPIs interaction is by targeting the K-RAS regulatory sites namely the nucleotide binding site and the switch II pocket (allosteric site).^299–302^

**Fig. 7:**
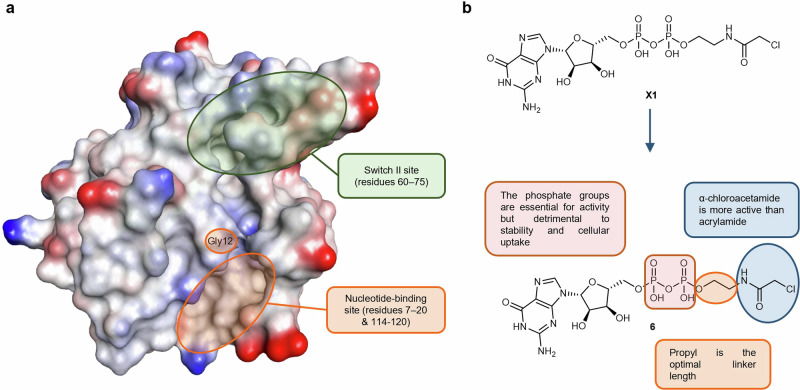
K-RAS Protein Structure And the K-RAS nucleotide binding site compound 6. **a** The K-RAS protein structure (PDB: 8FMI^476^) with the switch II region highlighted in green and the nucleotide-binding site highlighted in orange. **b** Chemical structure of compound **6** and key pharmacophoric features associated with its scaffold

The K-RAS protein comprises 188 residues that have been categorized into three distinct domains: the effector lobe (residues 1–86), the allosteric lobe (residues 87–166), and the hypervariable region (HVR) (residues 167–188). The nucleotide-binding site and the switch II pocket, which are located within the G domain of GTPase proteins, have been shown to be amenable to modulation by small molecule inhibitors. The guanine-nucleotide binding site, also known as the nucleotide-binding site, acts as the site where guanosine triphosphate (GTP) or guanosine diphosphate (GDP) bind to K-RAS.^303^ This binding switches K-RAS between its inactive (GDP-bound) and active (GTP-bound) forms, which causes the switch II pocket to fold, thereby allowing it to bind to and activate its effectors. The interactions between the switch I/switch II regions and GTP persist until the deactivation process is initiated by GTP hydrolysis. This event disrupts hydrogen bonds and releases the switch regions, causing the conformation to revert back to the inactive GDP-bound state.^304^ Alternatively, the hydrophobic switch II pocket located opposite to the nucleotide-binding site is characterized by undergoing conformational changes upon binding of K-RAS to GTP or GDP at the two flexible regions referred to as switch I (residues 32–38) and switch II (residues 60–75).^305,306^ Both the guanine-nucleotide binding site and the switch II pocket (Fig. 7a) play pivotal and interconnected roles in K-RAS function, impacting its activation state and interactions with downstream effectors within cellular signaling networks.^235^

## K-RAS nucleotide binding site small molecule inhibitors

The sub-nanomolar affinity of both GTP and GDP for RAS coupled with their abundant concentrations within cells has hampered efforts to develop small molecules that can inhibit RAS via competing with GTP and GDP at the guanine nucleotide binding site of the GTPases. However, the discovery and optimization of the covalent GTP mimic SML-8-73-1 (**6**) and its derivatives has been a successful strategy to overcome these challenges and present a viable option for K-RAS inhibition.^301,302^ The electrophilic α-chloroacetamide moiety of the GDP-mimetic **6** and its derivatives reacts with Cys12 following binding to K-RAS G12C.^301,302^

SAR analysis of **6** and its derivatives showed that the α-chloroacetamide moiety was more reactive toward Cys12 of K-RAS when compared to the acrylamide analog **X1**. Additionally, it was determined that a propyl linker provided an optimal distance between the β-phosphate and the reactive site of the α-chloroacetamide moiety which was further confirmed when attempts to shorten the linker resulted in a complete loss of activity. Cyclization of the linker to incorporate a pyrrolidine or cyclopentane ring maintained the activity although with lower affinity. Attempts to replace the phosphates significantly impacted binding affinity indicating that both phosphates may be essential for establishing high binding affinity with K-RAS. The chemical structure of **6** and its pharmacophoric features are summarized in Fig. 7b.

Unfortunately, **6** and its potent derivatives suffer from chemical instability due to the phosphate anhydride bond. Additionally, the charged nature of the phosphate groups of **6** and its derivatives rendered the compounds unable to cross the cell membrane which calls for further development if a viable clinical candidate is to be identified. The next step should be overcoming the stability and cellular uptake issues by replacing the phosphate groups with moieties that are capable of preserving binding activity whilst mitigating the liabilities.

## K-RAS allosteric site small molecule inhibitors

The switch II pocket (S-IIP) resides at the interface of the α2-helix (switch-II), α3-helix, and the core β-sheet of the RAS protein. The S-IIP presents an allosteric target distinct from the nucleotide-binding site and has emerged as a promising target for the development of mutant-specific K-RAS inhibitors. After the discovery of the S-IIP, various efforts were performed in order to identify covalent small molecule inhibitors capable of targeting the S-IIP which culminated in the FDA approval of sotorasib (**9**) in 2021 as the first targeted therapy for tumors harboring a K-RAS mutation.^16,307^ This was followed by the approval of adagrasib (**10**) in 2022 as a second distinct scaffold targeting the K-RAS G12C mutation in non-small cell lung cancer (NSCLC) patients with prior systemic therapy.^17,308^ While both adagrasib and sotorasib are successful examples of drug development targeting the allosteric S-IIP, the greater availability of reported sotorasib derivatives and their respective biological activity when compared to adagrasib offers a more comprehensive understanding of its SARs and optimization pathway.^307,309^

**Fig. 8:**
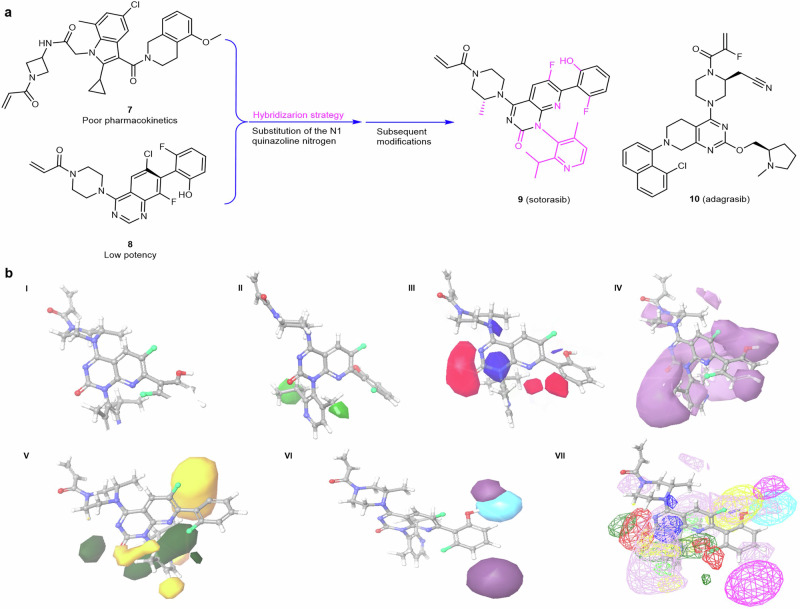
Sotorasib (9) design and development strategies. **a** Design strategy for identifying sotorasib (**9**) and chemical structure of adagrasib (**10**). **b** QSAR contour map of sotorasib (**8**): I. 3D structure of **9**; II. Steric contour map: Green regions indicate favorable steric interactions; III. Electrostatic contour map: Blue regions indicate positive electrostatic potential while red regions indicate negative electrostatic potential; IV. Hydrophobic contour map: Plum-colored regions indicate favorable hydrophobic interactions; V. Hydrogen bond acceptor map: Dark green regions indicate favorable hydrogen bond acceptor sites while yellow regions indicate unfavorable hydrogen bond acceptor sites; VI. Hydrogen bond donor map: Violet regions indicate favorable hydrogen bond donor sites while cyan regions indicate unfavorable hydrogen bond donor sites; VII. Combined contour map of all features: Overlaid mesh representation of all factors from (II) to (VI)

The development of sotorasib began with the discovery of the indole-based small molecule inhibitor **7** which was capable of occupying a previously unexploited cryptic pocket on the surface of K-RAS located at the S-IIP (Fig. 8a). However, **7** suffered from suffered from poor pharmacokinetic properties which was highlighted by low oral bioavailability and high clearance. To address these shortcomings, a hybridization strategy was performed in which key elements of **7** were combined with **8**, a quinazoline-based covalent S-IIP inhibitor which suffered from low K-RAS potency. A range of modifications were explored as an approach to increasing the potency of the new hybrid and to improve its pharmacokinetic and pharmacodynamic profile. These optimization efforts involved the addition of an isopropylphenyl group capable of occupying the cryptic pocket of S-IIP as well as the substitution of the C_2_ piperazine and replacement of the C_7_ fluorophenol. While these modifications led to increased inhibitory activity, the hybrid molecules suffered from low inhibitory solubility and poor membrane permeability.

Rational drug design efforts revealed that incorporating a nitrogen atom into the quinazolinone ring resulted in an azaquinazolinone that demonstrated significantly improved membrane permeability and inhibitory activity. However, the azaquinazolinone structure resulted in atropisomerism which is axial chirality around the biaryl bond introducing unwanted rotational configurations. This challenge was resolved by avoiding atropisomerism altogether via employing symmetrically substituted cryptic pocket-binding elements. These efforts led to the successful discovery and development of sotorasib (**9**) highlighting how rational design efforts can lead to a viable clinical candidate as well as emphasizing the considerable potential of PPIs.

Taking advantage of the available biological data for the various derivatives of sotorasib (**9**), we attempted to identify the 3D quantitative structure–activity relationships (3D QSARs) associated with **9** and its analogs to elucidate the relationship between the K-RAS inhibitory activity exhibited by the compounds and the key structural features. The field-based QSAR module of the Maestro Schrodinger program (version 2021.2) was employed according to the procedures of a previous study to analyze the QSARs based on the pIC_50_ values of sotorasib and its derivatives.^310^

The QSAR model displayed an *R*^2^ of 0.84 and *Q*^2^ of 0.76 which gives confidence in the predictive abilities of the model and its results. Analysis of the QSAR model results revealed that the sotorasib (**9**) scaffold readily accommodated bulky substituents on the pyridine ring which indicates flexibility in this region. Moreover, all four core elements, the piperazine, the fluorophenol, the azaquinazolinone, and the pyridine ring, were predicted to tolerate hydrophobic substituents which aligns with the largely hydrophobic nature of the S-IIP of K-RAS. Furthermore, the QSAR model predicted that both N_12_ of the azaquinazolinone and N_7_ of the piperazine benefit from positive electrostatic substituents while electrostatic substituents on the fluorophenol and the azaquinazolinone carbonyl were predicted to be unfavorable. Additionally, substituting the pyridine ring with hydrogen bond acceptors was predicted to enhance the K-RAS inhibitory activity. Conversely, the fluorophenol ring was predicted to tolerate hydrogen bond donor substituents. These findings identify the relationship between the pharmacophoric features **9** and its K-RAS inhibitory activity which provides a roadmap for future attempts at improving the potency. The QSAR contour map based on the K-RAS inhibitory activity of sotorasib (**9**) and its derivatives is illustrated in Fig. 8b.

The successful development and FDA approval of sotorasib (**9**) exemplify the power of hybridization strategies to deliver clinically viable drug candidates. Additionally, the FDA approval of **9** not only underscores the potential of hybridization strategies but also reinforces the validity of using small molecules to target PPIs. Moreover, the QSAR analysis of **9** and its derivatives highlight how optimization of small molecules can be carried out by exploiting existing data to rapidly identify and visualize pharmacophoric sites that can be used for lead optimization.

### Anti-inflammatory and immunomodulatory PPI modulators

Inflammation is a key response of the body’s defense mechanism which serves as the first line of defense against invading pathogens and cellular damage.^311–313^ The inflammatory system is a delicate and intricate network that relies on a well-orchestrated response mediated by the immune system. This response is crucial for maintaining tissue health and homeostasis.^314,315^ Proper functioning of the inflammatory system ensures that harmful stimuli such as pathogens or damaged cells are effectively addressed while minimizing damage to healthy tissue. However, inflammation becomes detrimental when it persists or misdirects its attack which occurs when the immune system mistakenly targets the body’s own tissues (autoimmunity) or when the response to an initial trigger becomes uncontrollable.^316,317^ In such cases, the attempts of the immune system to eliminate the harmful stimuli lead to chronic inflammation which is a hallmark of many diseases. The inflammatory response is regulated by many PPIs which are essential for mediating the immune response and maintaining homeostasis. In this section, we will focus on gp130/IL-6 and 14-3-3 interactions and their modulation.

#### Gp130/IL-6 inhibitors

Cytokines are signaling proteins with short lifespans which are involved in cell communication via acting through autocrine, paracrine, and endocrine signaling pathways.^318–321^ Among the different cytokines, the IL-6 family of cytokines stands out for its reliance on a common signaling subunit, glycoprotein 130 kDa (gp130).^322–329^ The Gp130 receptor contains three domains: an extracellular region for ligand binding, a transmembrane segment anchoring the receptor to the cell membrane, and a cytoplasmic domain responsible for intracellular signaling. gp130 Serves as the signal-transducing subunit for the IL-6 family which when bound to a ligand such as IL-6 activates the JAK signaling pathway leading to triggering a cascade of events that ultimately lead to the activation of STAT transcription factors.^330–332^

While gp130 is found in most cells, its presence alone is not enough for a cell to respond to IL-6 family cytokines. The interaction of the IL-6 family cytokines with their specific partner receptor subunits is crucial for eliciting a cellular response. For example, IL-6 and IL-11 need to bind to their respective non-signaling α receptors (IL-6Rα and IL-11Rα) before interacting with gp130. The resulting IL-6/IL-6Rα complex then activates gp130 homodimers. Meanwhile, other members of the cytokine family, such as the ciliary neurotrophic factor (CNTF) and Oncostatin M (OSM), require a heterodimeric complex to initiate signaling. For example, CNTF signals through a gp130-LIFR heterodimer, involving both gp130 and another signaling receptor, LIFR.^333–336^

**Fig. 9:**
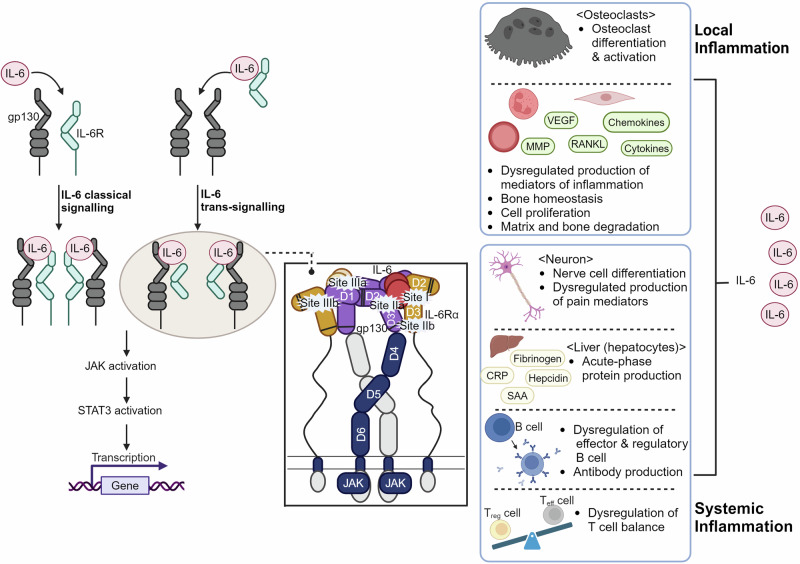
Comprehensive overview of IL-6 signaling pathways, associated inflammatory response, and schematic representation of IL-6/IL-6Rα/gp130 complexes. Created by Biorender

Understanding the intricate interplay between gp130 and the IL-6 family of cytokines as well as its ability to differentiate between the different cytokines necessitates a detailed understanding of the gp130 structure. The extracellular region of gp130 (Fig. 9) consists of an N-terminal immunoglobulin (Ig)-like domain, a cytokine-binding module (CBM), and three fibronectin type III (FNIII)-like domains. Each cytokine binds to gp130 in a distinct manner; for example, IL-6 possesses three distinct receptor-binding sites. Site I interacts with the CBM of IL-6Rα while sites II and III involve specific regions on the gp130 homodimer. The CBM of gp130 interacts with site II and the Ig-like domain interacts with site III. Neither IL-6 nor the soluble form of IL-6Rα (sIL-6Rα) can bind gp130 with significant affinity on their own. However, the IL-6/sIL-6Rα complex binds gp130 with high affinity (picomolar range). This complex essentially provides two binding interfaces where each binding interface is realized by contributions from both IL-6 and IL-6Rα, acting as composite binding sites for gp130. These composite binding sites explain why only the IL-6/IL-6Rα complex can activate gp160.^337–340^

The CBM of gp130 binds to a specific interface (site IIa-IIb) on one IL-6/IL-6Rα molecule. Two such 1:1:1 complexes (IL-6/IL-6Rα/gp130) convene through interactions between the Ig-like domain of gp130 (D1) and another interface (site IIIa-IIIb) on a separate IL-6/IL-6Rα molecule. This interaction leads to the formation of a final hexameric complex with a 2:2:2 stoichiometry (two IL-6 molecules, two IL-6Rα molecules, and two gp130 molecules). The presence of two independent ligand-binding sites on gp130 is crucial for this higher-order complex formation, which is essential for signal transduction. The proinflammatory activities of IL-6 are mediated by IL-6 trans-signaling *via* the sIL-6R, whereas the protective and anti-inflammatory activities of IL-6 are mainly executed via the membrane-bound IL- 6R (classic signaling).^341–344^

##### gp130/IL-6 small molecule inhibitors

The development of small-molecule inhibitors for gp130 has become a significant area of research over the last two decades. These efforts have focused primarily on the shared binding site for IL-6 and IL-11 located in the D1 domain within the extracellular domain of gp130. Targeting of this common binding site within gp130 has led to dual inhibitory effects on both IL-6 and IL-11 activation. Studies have revealed that these small-molecule inhibitors interact with specific “hot spots” on the D1 domain at three main “sites”: Leu57, Trp157, and an extra binding site. Recent perspectives have examined the potential of gp130 as a drug target for small molecule development as well as detailed analyses of the SARs and binding potential of small molecule inhibitors targeting gp130.^99,345^

##### IL-6/IL-6Rα/gp130 mAbs

While there are no FDA-approved gp130/IL-6 small molecule inhibitors, significant progress has been made in developing antibody-based therapeutics.^99,346–348^ Currently, the FDA has approved four IL-6R mAbs for clinical use: tocilizumab, siltuximab, sarilumab and satralizumab in 2010, 2014, 2017, and 2020, respectively.^15,349–351^ Among the four mAbs, tocilizumab was the first to be approved and paved the way for the subsequent development of the other mAbs. Tocilizumab is a humanized recombinant monoclonal antibody which is produced by grafting the complementarity-determining regions (CDRs) of a murine anti-human IL-6R antibody onto a human IgG1 framework.^352–354^ Tocilizumab is capable of targeting both the IL-6 classic and trans-signaling pathways making it a powerful therapeutic agent. Tocilizumab exerts its IL-6 inhibitory action by directly binding to the IL-6R receptor, thereby preventing IL-6 from binding to gp130. By preventing this complex formation, tocilizumab effectively blocks IL-6 signaling in cells that only express gp130. Additionally, tocilizumab has been reported to cause the dissociation of pre-formed IL-6/sIL-6R complexes which disrupts existing signaling and further strengthens its inhibitory action on the IL-6 pathway.^13,355–359^

Extensive clinical trials have demonstrated the efficacy of tocilizumab in rheumatoid arthritis (RA) patients which has led to its approval for treating moderate to severe RA in various countries, including the USA and the EU.^360^ It has also been approved for the treatment of giant cell arteritis, systemic sclerosis-associated interstitial lung disease, polyarticular juvenile idiopathic arthritis, systemic juvenile idiopathic arthritis, and cytokine release syndrome. In June 2021, the FDA granted it emergency use authorization (EUA) for hospitalized pediatric COVID-19 patients receiving corticosteroids. Studies have shown that adding tocilizumab to standard COVID-19 treatment regimens significantly reduces mortality rates and the need for hospitalization or ventilation.^361–363^ The diverse therapeutic applications of these IL-6 inhibitors underscore the potential of targeting gp130/IL-6 signaling as a broad strategy for managing inflammatory diseases.

#### PPI modulators

14-3-3 proteins are eukaryotic adaptor proteins involved in many cellular processes such as cell-cycle control, signal transduction, protein trafficking, and apoptosis. By binding to other proteins, 14-3-3 can assist in protein folding, protein localization, and stimulation or inhibition of other PPIs. It has been demonstrated that 14-3-3 has over 200 structurally diverse and functionally different interacting partners.^364^ Thus, 14-3-3 proteins constitute an important family of regulatory proteins that exert a significant impact on the regulation of inflammatory processes.^365–367^ 14-3-3 Proteins bind to partner phosphorylated proteins to exert downstream effects including protein degradation, membrane localization and nuclear exclusion.^368^ Additionally, 14-3-3 proteins are involved in regulating transcription factors and immune response effectors. At the molecular level, integral elements of the inflammatory process such as pattern recognition receptors, protease-activated receptors, and cytokines undergo phosphorylation and subsequent recognition by 14-3-3 proteins. Disruption of the recognition processes between 14-3-3 proteins and their respective partners has been observed to result in clinical syndromes. Additionally, abnormal levels of 14-3-3 proteins contribute to undesirable immune responses and chronic inflammatory conditions.^369,370^

**Fig. 10:**
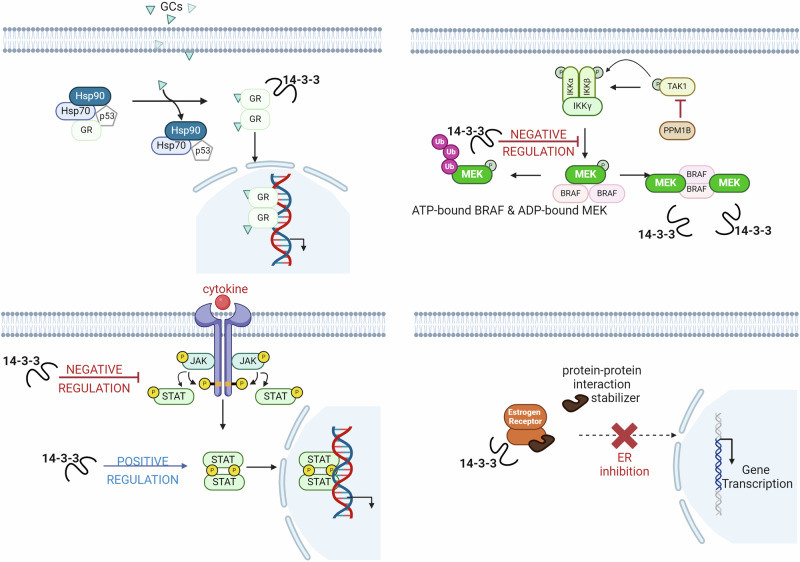
Regulation of Inflammatory Transcription Factors by 14-3-3 Proteins. Key interactions between 14-3-3 isoforms and target transcription factors are illustrated to highlight the functional consequences of these interactions on inflammatory gene expression and signaling pathways. Created by Biorender

The 14-3-3 family is comprised of seven isoforms designated as β, ε, γ, η, σ, τ, and ζ that share a high sequence similarity, especially in the amphipathic binding groove. However, the different 14-3-3 isoforms have been shown to possess specific roles and differential tissue expression levels. The 14-3-3 proteins primarily function as heterodimers and bind to proteins containing phosphorylated serine/threonine residues, thereby regulating various transcription factors involved in the inflammatory response (Fig. 10).^371–373^ These transcription factors that are subject to 14-3-3 modulation include the glucocorticoid receptor (GR), peroxisome proliferator-activated receptors (PPARs), Janus kinase-signal transducer and activator of transcription protein (JAK-STAT), and the estrogen receptor (ER).^374–376^ Additionally, 14-3-3ζ was reported to be an endogenous suppressor of inflammatory arthritis.^366^ Consequently, the development of PPI modulators targeting 14-3-3 holds promise for treating chronic inflammatory diseases linked to aberrant 14-3-3 levels. Likewise, since many of the 14-3-3 binding partners are typically considered undruggable proteins, 14-3-3 PPIs modulators are promising strategies for modulating these targets.

##### 14-3-3 molecular glues

Designing molecular glues with high selectivity and good PK properties for specific PPIs is a significant challenge due to the common interface shared by many PPI heterodimers. For example, both ERα and GR share the same binding pocket on the 14-3-3 protein. Optimization of **11**, a known Fusicoccin A (FC-A) based compound, has led to the development of a series of molecular glues with the ability to selectively modulate either the 14-3-3/ERα or 14-3-3/GR PPIs.^377,378^ While **13** exhibited weak stabilization of the 14-3-3/GR interaction, its racemic mixture lacked selectivity towards either GR or ERα. Intriguingly, although the (*S*)-enantiomer exhibited weak activity, it displayed remarkable selectivity for the 14-3-3/GR interaction. This selectivity held true for other FC-A derivatives, indicating the importance of stereoisomerism on compound selectivity.

**Fig. 11:**
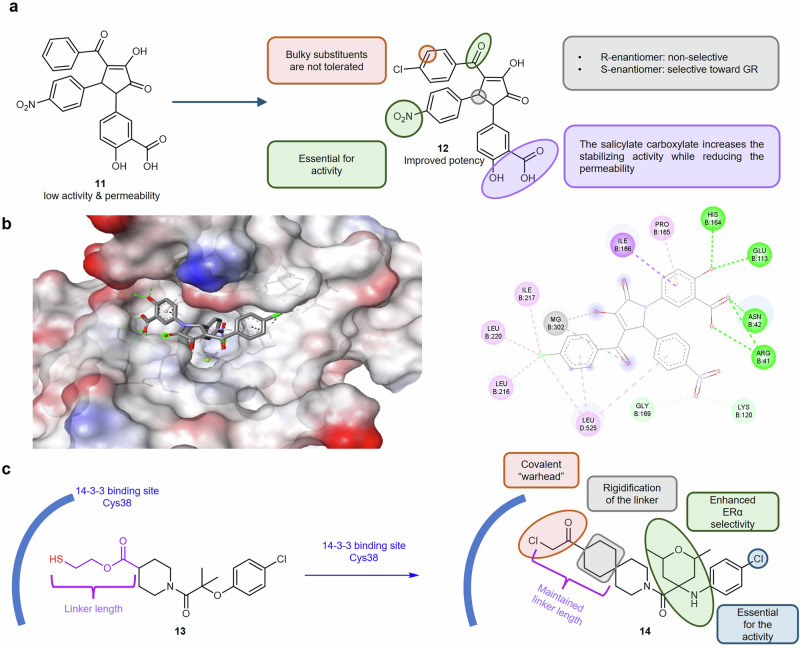
14-3-3 molecular glues. **a** SAR associated with FC-A-based molecular glues. **b** Molecular docking of **12** in complex with 14-3-3 (PDB: 8A9G). **c** Insights into the optimization of **13** to **14**

Rational optimization of **11** revealed that the aroyl moiety (R_1_) exerts a significant influence on the selectivity of the synthesized molecular glues. Introducing a 4-Cl group on the R_1_ moiety yielded the most potent derivative, **12**. X-ray cocrystallography revealed that **12** interacts within the FC-A pocket through polar interactions and hydrogen bonds (Fig. 11b). In the 14-3-3ζ/GR complex, a fully hydrated Mg^2+^ ion was observed chelated by the vinylogous carboxylate moiety of (*R*)-**12**, potentially pre-organizing its conformation for optimal binding. Removing this carbonyl group disrupted metal chelation and likely caused a mismatch between the solution and binding conformations. This finding indicates that the carboxylate moiety is essential for the stabilizing activity.

Further SAR analysis identified the 3-position of the R_1_ phenyl ring as a sterically congested region which indicates that bulky substitutions at this position are not tolerated. Removal or reduction of the NO_2_ moiety of the R_2_ ring led to significant decrease in the 14-3-3 stabilizing activity. While this series of 14-3-3 small molecule stabilizers exhibits considerable potential due to a promising selectivity profile and potency, two major drawbacks are apparent. The first major concern is that the NO_2_ moiety of the R_2_ ring has been associated with mutagenicity and genotoxicity if metabolized. In addition, while **12** showed the highest potency, it displayed poor membrane permeability which highlights opportunities for future modifications. Figure 11a illustrates the collective SARs associated with the FC-A-based molecular glues as well as the binding pattern of the most potent derivative, **12** with the 14-3-3 protein.

Despite these challenges, this approach demonstrates the significant impact of regioisomerism and subtle changes in functional groups on both the selectivity and bioactivity, respectively. Furthermore, chirality and regioisomerism should not be perceived as obstacles to be avoided solely because of the challenges associated with separating different isomers. Instead, they should be regarded as opportunities to tackle selectivity issues that might otherwise be challenging to address. Moreover, this series of 14-3-3/GR molecular glue holds promise as the challenges faced by this series can be addressed through established drug design optimization approaches. For example, a potential solution to address the membrane permeability issue observed with **12** could involve the development of prodrugs through esterification of the carboxyl group with specific labile esters that may enhance its permeability. These esters are readily cleaved by enzymes in the body which would lead to the release of the active compound at the target site.^379,380^

Much like PPI inhibitors, molecular glues can bind to their target site in either a reversible or irreversible (covalent) manner. A study involving the careful integration of molecular docking, X-ray crystallography and rational drug design was successful in the identification of potent and specific covalent 14-3-3/ERα small-molecule stabilizers.^381^ This approach holds considerable promise due to the direct link between ERα and breast cancer development and cell proliferation. 14-3-3 Proteins are responsible for the suppression of the transcription activity of ERα by binding to its extreme C-terminus (Fig. 10). Therefore, stabilizing the 14-3-3/ERα is a viable therapeutic strategy.

The study involved the optimization of **13**, a nonselective 14-3-3 stabilizer (Fig. 11c), by integrating the structural information obtained from molecular docking, X-ray cocrystallography, and rational drug design. This optimization strategy involved substituting the reversible disulfide linkage with irreversible electrophiles, such as chloroacetamide. Next, in order to enhance the selectivity of the designed molecular glues for ERα, anilines were introduced in place of ethers, and cyclic aliphatic rings replaced the gem-dimethyl group at the specified position (Fig. 11c). The improved selectivity was attributed to the aniline’s involvement in a water-mediated hydrogen bond with the terminal carboxyl group of ERα. Simultaneously, the enhanced stabilization observed with the gem-dimethyl moiety was attributed to its occupancy of the hydrophobic pocket of 14-3-3, as observed in the peptide interaction interface during molecular docking simulations. The significance of the p-Cl group in establishing a halogen bond for stabilizing activity was confirmed through biological testing, where the removal or alteration of different groups led to a loss or reduction in activity. Additionally, the observed 3.5 Å distance indicated that larger halogens would induce steric hindrance, while smaller substituents would fail to interact optimally with Lys122. The SAR of the designed molecular glues is summarized in Fig. 11c overlayed over the most potent compound, **14**.

This study is a prime example of the successful integration of multiple drug design strategies, where X-ray crystallography-based screening identified the original fragment. Subsequent optimization of the fragment involved observing the molecular structure of the 14-3-3/fragment complex and identifying the possible empty pockets proximal to the binding site to increase affinity. This involved the modification of **13** by replacing the gem-dimethyl group with a cyclic motif designed to more optimally occupy this pocket. Additionally, X-ray cocrystallography was employed to identify the best orientation for binding which inspired the rigidification of the linker moiety, thereby stabilizing the compound in a preferred conformation.

##### Peptidomimetic 14-3-3 inhibitors

**Fig. 12:**
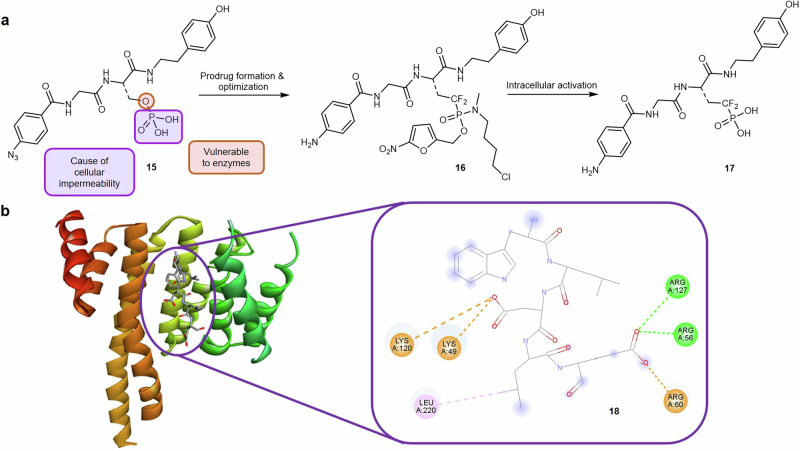
14-3-3 inhibitors peptides and peptidomimetic inhibitors. **a** The figure illustrates the discovery of the peptidomimetic **15**, followed by the design of a prodrug (**16**) to enhance cell entry. Prodrug **16** is converted inside the cell into its active form **17**, retaining the essential functional group. **b** The crystal structure of **18** in complex with 14-3-3 (PDB: 1A38) and their key interactions illustrated by Discovery Studio Visualizer V.2023

Significant effort has been made toward the development of peptidomimetics that can disrupt phosphorylation sites recognizing phosphotyrosine or phosphoserine/threonine motifs which mediate a range of protein-protein interactions, including those involving 14-3-3 proteins.^382–386^ The first peptidomimetic (**15**, Fig. 12a) capable of disrupting 14-3-3 proteins was discovered using the SMM technique where diverse fragments were constructed based on a known optimal 14-3-3 binding sequence.^387^ The resulting fragments were then displayed on a glass slide where their binding affinity was measured by their interaction with a fluorescently labeled 14-3-3 protein. This screening process identified five promising fragments with strong binding from which **15** was designed by strategically combining the fragments leading to a molecule with a potent inhibitory effect on the 14-3-3σ isoform, IC_50_ = 2.6 μM. Unfortunately, **15** suffered from poor cell permeability due to the presence of the negatively charged phosphate group which was essential for targeting the phosphoserine/threonine motifs but impeded its ability to traverse the lipophilic cell membrane. Moreover, the phosphate group in **15** was vulnerable to breakdown by enzymes within the cell.^238,387–389^

Subsequent efforts led to the design of the prodrug **16** which overcame the two main issues of **15**. In **16**, a difluoromethylene linker replaced the phosphate-to-serine connection which protected the peptidomimetic from phosphatases while preserving the necessary electrical charge for targeting the 14-3-3 protein. Additionally, the phosphonate group of **15** was neutralized by reacting the two phosphonate oxygens with 4-chloro-N-methylbutan-1-amine and nitro-furfuryl groups. Once **16** was inside the cell, enzymatic-mediated cleavage of the nitrofurfuryl group and subsequent intracellular cyclization of an intermediate produced an intermediate prone to spontaneous hydrolysis which afforded the biologically active phosphonate peptidomimetic **17**.^390^

##### Peptide based 14-3-3 inhibitors

The discovery and optimization of the peptide-based 14-3-3 inhibitor R18 (**18**) highlighted the potential of peptides to modulate 14-3-3 proteins.^391^ R18 (**18**) is a composed of 20 residues with a high affinity for the canonical amphipathic binding groove of 14-3-3 proteins. Crystallographic analysis (PDB: 1A38^392^) revealed two hydrogen bonds formed between a glutamic acid side chain in **18** and Arg56 and Arg157 residues on 14-3-3. Additionally, robust salt bridges were established between **18** and Arg60, Lys49 and Lys120 amino acid residues of 14-3-3 (Fig. 12b). Lastly, the presence of leucine residues in **18** facilitated the formation of a crucial hydrophobic interaction with the Leu220 in the complementary regions on the 14-3-3 protein which further stabilized the **18**/14-3-3 complex. Notably, **18** disrupted both the phosphorylation-dependent and -independent 14-3-3 interactions which imply its potential in modulating a broader spectrum of cellular signaling pathways compared to the aforementioned peptidomimetics **15**-**17** which focus on the phosphorylation-dependent region. Subsequent efforts led to the design of an extended peptide which comprised of 64 amino acids and incorporated two **18**-like motifs. Functional assays of the extended peptide demonstrated superior inhibitory activity against 14-3-3 PPIs and the ability to induce apoptosis in cancer cells. In vivo models corroborated these findings with the extended peptide significantly enhancing the efficacy of cisplatin and demonstrably suppressing tumor growth. The development of **18** underscores the therapeutic potential of targeting 14-3-3 PPIs using peptides and developing targeted therapeutics.^393,394^

### Antiviral inhibitors of PPIs

One novel application of PPI modulators is the inhibition of viral infections by targeting the interactions between viral and host proteins or between viral proteins.^50,395^ Viruses are simple organisms with sophisticated mechanisms of hijacking host cell processes to ensure reproduction and propogation.^396^ Since the viral reproduction process typically involves a series of PPIs between viral and host cell proteins, PPI inhibitors can disrupt these interactions to inhibit viral replication.^397^

Antiviral PPI inhibitors may offer several advantages over therapies that target viral enzymes and can be more resistant to mutations that engender drug resistance.^398,399^ These advantages stem from several factors among which is their ability to target large and flat surfaces on proteins. This property of PPIs can render them less susceptible to resistance development when compared to traditional inhibitors that target specific active sites. Additionally, viruses may have functional redundancy in their enzymatic activities which provides aid in overcoming inhibitors through mutations. Conversely, disrupting critical PPIs can be more detrimental to the virus, as these interactions are often integral to the viral lifecycle and less likely to have redundant pathways.^400,401^ Another advantage of antiviral PPI inhibitors over traditional therapeutics lies in their ability to disrupt essential interactions that are critical for viral replication.^402,403^ Studies have shown promise in targeting PPIs for diverse viruses like HIV-1, coronaviruses, and influenza virus with varying degree of success.^404–411^

One of the promising applications for antiviral PPI inhibitors is for the treatment of HIV-1 which subtends acquired immunodeficiency syndrome (AIDS), a global health crisis which affects over 38 million people worldwide and is the result of the progressive depletion of T lymphocytes.^395,412^ HIV-1 exhibits high infectivity and transmission rates^413,414^ and infects target cells in a series of well-defined steps. First, the viral envelope glycoprotein 120 (gp120) attaches to the cellular CD4 receptor which triggers conformational changes in gp120, enabling its subsequent binding to a coreceptor, either CCR5 or CXCR4, on the target cell surface. Coreceptor binding induces a second conformational rearrangement that liberates the viral fusion protein which is embedded in the N-terminus of gp41. This final step leads to interaction with the host cell membrane and fusion between the viral and cellular membranes.^415,416^ Given their crucial roles in this process, gp120, CD4, coreceptors (CCR5 and CXCR4), and gp41 represent promising targets for therapeutic intervention.

**Fig. 13:**
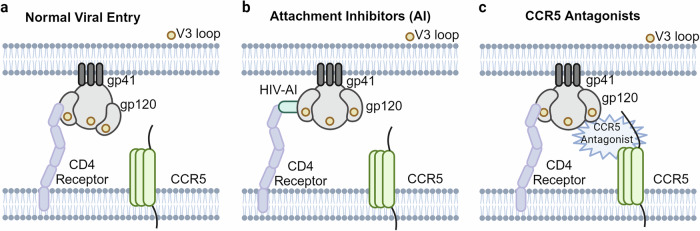
Mechanisms of HIV-1 entry and potential sites for therapeutic intervention. **a** Normal HIV-1 entry pathway. Inhibition of HIV-1 by attachment inhibitors (**b**) and CCR5 antagonists (**c**). Created by Biorender

Antiviral agents that disrupt any step of the HIV-1 entry process are classified as entry inhibitors.^417^ These drugs are particularly valuable for heavily treatment experienced (HTE) individuals who have limited treatment options due to the acquisition of extensive drug resistance.^418,419^ Typically used in combination with other antiretroviral drugs as part of highly active antiretroviral therapy (HAART) regimens, entry inhibitors prevent viral entry by targeting specific steps in the process (Fig. 13). There are three main categories of entry inhibitors based on their mechanism of action: 1) attachment inhibitors (AIs) which block the initial encounter between virus and host cell either by binding to host CD4 proteins or virus gp120, 2) CCR5 or CXCR4 antagonists which bind to the respective coreceptors to interrupt association with gp120 and the liberation of gp41 and 3) fusion inhibitors which interact directly with gp41 to interfere with the cell membrane fusion process. The success and potential of antiviral PPI inhibitors is highlighted by the FDA-approval of the AI temsavir, administered as its prodrug fostemsavir, and maraviroc, a CCR5 antagonist.^420,421^

When compared to traditional antiviral therapeutics, antiviral protein-protein interaction (PPI) inhibitors offer several advantages. Traditional antivirals typically target viral enzymes or structural proteins, often leading to the rapid emergence of drug-resistant viral strains due to high mutation rates.^422,423^ In contrast, PPI inhibitors disrupt the interactions between viral proteins and their host cell counterparts which are generally more conserved and less prone to mutations.^424^ This reduces the likelihood of resistance development. Additionally, targeting PPIs can interfere with multiple stages of the viral lifecycle leading to potentially increasing therapeutic efficacy.^425,426^ PPI inhibitors can also be designed to modulate host factors essential for viral replication, thereby broadening the scope of antiviral targets and potentially offering cross-protection against diverse viruses.^402,427^ Moreover, advancements in structural biology and computational chemistry have enhanced the design and optimization of PPI inhibitors, improving their specificity and reducing off-target effects. These attributes make antiviral PPI inhibitors a promising alternative to traditional antiviral therapies, offering the potential for more durable and broadly effective treatments.

#### HIV-1 small molecule PPI inhibitors

The HIV-1 AI temsavir (**20**) binds to a specific region on the HIV-1 envelope gp120 adjacent to the CD4 binding pocket in a fashion that pushes the β20-β21 and Trp_427_ loops into the CD4 binding pocket^428,429^ thereby stabilizing the viral envelope protein in a conformation unable to bind to CD4 but which interestingly, is targeted by some broadly neutralizing antibodies (bNAbs) including V_1_/V_2_-directed bNAbs.^430,431^ The prototype for **20** was discovered with a HTS using a non-infectious HIV-1 pseudovirus assay to screen ~100,000 compounds from the Bristol Myers Squibb corporate library which surfaced the promising lead compound **19** which displayed an EC_50_ value of 153 nM.^432–434^ Extensive optimization efforts ultimately yielded **20** which exhibited significantly improved potency (EC_50_ = 0.1 nM) and a broader HIV-1 inhibitory spectrum.^435–437^ However, the poor aqueous solubility of **20** resulted in an inadequate pharmacokinetic performance, a deficiency overcome by exploiting the phosphonooxymethyl prodrug found in fostemsavir (**21**), the marketed form.

**Fig. 14:**
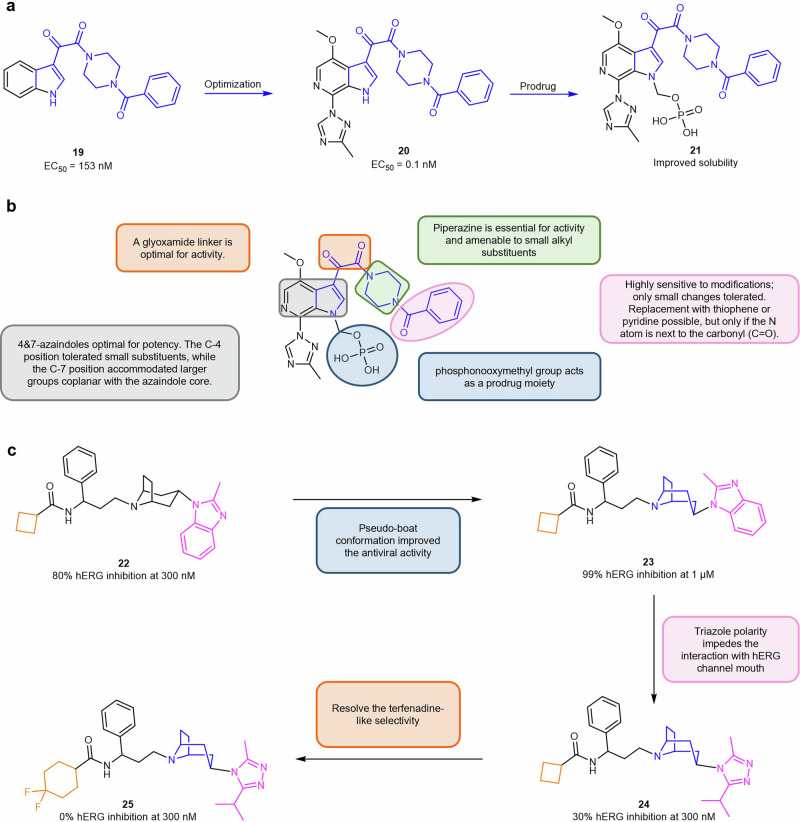
HIV-1 small molecule PPI inhibitors design strategies. **a** Milestones in the development of **21**. **b** SARs associated with **20**. **c** The optimization journey of maraviroc (**25**) from a screening hit to a clinical candidate

The SARs associated with **20** (Fig. 14) indicate that the azaindole core of **20** was derived from the indole lead as an approach to enhancing physicochemical properties, with 4 and 7-azaindoles optimal for potency, the 5-azaindole poor and the 6-azaindole a compromise that allowed simultaneous substitution at C-4 and C-7, the most important sites for modulating potency. Decoration at C-4 was restricted to small substituents with methoxy, fluorine and chlorine optimal. In contrast, C-7 was tolerant of much larger substituents although there was a requirement for coplanarity with the azaindole core. At C-3, a glyoxamide moiety was optimal with the oxygen atom of the C=O distal to the azaindole core engaging with the backbone N-H of Trp427 in a H-bonding interaction. The piperazine heterocycle was essential to project the benzamide moiety in a conformation that facilitates π-interactions with the indole of Trp427 and the phenyl ring of Phe382 and would tolerate only small alkyl substituents. The terminal phenyl ring was highly sensitive to modification with only small changes tolerated although it could be replaced with thiophene and a pyridine ring provided that the *N* atom was adjacent to the C=O.

CCR5 antagonists represent the second PPI strategy that has been proven to be effective in targeting HIV-1 infection. The chemokine receptors CCR5 and CXCR4 are critical coreceptors that facilitate the entry of HIV-1 into host cells, with the former the method of entry early in infection whilst the latter becomes more dominant late in chronic infection.^438,439^ Initially, the viral protein gp120 binds to the CD4 receptor on the host cell surface which triggers a conformational change in gp120 to expose the chemokine-coreceptor binding sites. The subsequent interaction of gp120 with either CCR5 or CXCR4 determines the viral tropism with R5 viruses utilizing CCR5 for entry while X4 viruses preferentially exploit CXCR4. R5X4 viruses, although less common, have the ability to bind to either coreceptor. In patients with advanced infections, HIV-1 evolves to exploit CXCR4 which correlates with declining CD4^+^ T cell counts and accelerated progression to AIDS. CCR5 antagonists target a specific pocket within the receptor transmembrane domain which induces conformational changes that interfere with the gp120-CCR5 interaction, thereby preventing the entry of HIV-1 into host cells. However, a challenge to this therapeutic strategy is the substantial variability observed in HIV-1 gp120 although patients heterozygous for a Δ32 deletion in CCR5, which introduces a premature stop codon, exhibit slower disease progression whilst homozygotes are resistant to infection.^12,440–442^

The FDA approval of maraviroc (**25**) (Fig. 14c) highlighted the feasibility of CCR5 antagonism as a therapeutic approach to HIV-1 treatment. The development of **25** started with the identification of **22** from an HTS campaign.^443,444^ Subsequently, **23** was synthesized by inducing a pseudo-boat conformation in the piperidine ring of the parent **22**. This conformational adjustment effectively reduced the 1,3-diaxial strain between the tropane bridge and the benzimidazole moiety. The synthesized isomer **23** exhibited improved antiviral activity which highlights the impact of spatial arrangement on potency. Subsequent efforts focused on mitigating the compound’s hERG channel inhibition by adding polar amide substituents which led to the replacement of the benzimidazole core with a triazole featuring a simple methyl group (**24**). Compared to **23** (99% inhibition at 1 µM), **24** displayed a significant reduction in hERG inhibition, reaching only 30% inhibition at a higher concentration (300 nM). The reduction in hERG inhibition was attributed to the polarity of the triazole moiety, which was deemed crucial for diminishing interactions with channel residues. This observation suggests that incorporating polarity into amide substituents may yield similar benefits in reducing hERG affinity. However, **24** suffered from a similar potential QTc prolongation profile to that of terfenadine, a drug withdrawn due to this kind of cardiotoxicity. This raised safety concerns about drug-drug interactions, particularly with HIV-1 treatment regimens which depend fundamentally on the use of combination therapy that can include CYP 450 inhibitors such as ritonavir. Further optimization replaced the cyclobutyl amide with a 4,4-difluorocyclohexyl group to arrive at maraviroc (**25**) which was devoid of hERG channel binding at 300 nM while maintaining potent antiviral activity (EC_90_ = 2 nM).^445–448^

#### HIV-1 mAbs

Ibalizumab (TNX-355) is an FDA-approved humanized IgG4 monoclonal antibody that targets the CD4 receptor which disrupts the HIV-1 entry process.^449–451^ While traditional antibodies inhibit the process of gp120 binding to CD4, studies have shown that ibalizumab possess a unique mechanism of action in which the mAb induces post-binding conformational changes which prevent the CD4-bound gp120 from interacting with the CCR5 or CXCR4 co-receptors. This mode of action does not affect the MHC-II function of CD4 leading to increased tolerability and lack of immunosuppression.^452–454^

Ibalizumab’s unique mode of action was confirmed by structural analyses which revealed that the mAb bound to CD4 on the side opposite the binding sites for gp120 and MHC-II. Further analysis showed that the key binding residues L96, P121, P122, Q163 in domain 2, and E77, S79 in domain 1 cluster away from the viral and immune binding regions. Mutagenesis experiments identified the essential residues for ibalizumab binding on human CD4 as E77, S79, P121, P122, and Q163. These findings support the importance of domains 1 and 2 for binding while highlighting potential conformational changes due to amino acid substitutions. Interestingly, residues previously thought to be crucial appear to be less important, suggesting that different epitope mapping methods may influence results.^452,455,456^ The distinct epitope of ibalizumab explains its lack of interference with MHC-II function.

Phase 3 trials of ibalizumab demonstrated its safety, tolerability, and potential for monotherapy in patients suffering from multidrug resistance and revealed significant antiviral activity (>1 log_10_ viral load reduction) with weekly intravenous dosing.^457^ The long half-life of ibalizumab combined with its unique mechanism of action reduces the risk of cross-resistance and offers promise as a viable therapeutic solution to multidrug resistance.^458^

#### Peptide-based PPI inhibitors of HIV-1 infection

A number of studies have been performed with the objective of developing peptides capable of disrupting the formation of the six-helix bundle (6-HB) within gp41 that is a critical step in HIV-1 infection. These peptides are designed to mimic the N-terminal heptad repeat (NHR) and C-terminal heptad repeat (CHR) regions of gp41. The peptides competitively bind to the exposed NHR or CHR in the pre-hairpin conformation of gp41 which prevents the formation of the 6-HB and ultimately blocks the fusion of virus and cellular membranes.^459,460^ Among the various efforts undertaken to develop peptide-based HIV PPI inhibitors, the development of sifuvirtide provides valuable lessons for future PPI inhibitor design.^461–463^

Enfuvirtide (T20/Fuzeon^TM^) is a 36-amino acid CHR-derived peptide which in 2003 was the first HIV-1 fusion inhibitor approved for clinical use.^464^ However, its effectiveness was hampered by the development of resistance as well as fibrillation (aggregation), a common problem with peptide-based therapeutics.^465–467^ One approach to address this limitation involved the use of cucurbit[7]urils (CB[7]), a water-soluble macrocycle that can specifically bind to the C-terminal Phe residue of enfuvirtide leading to the modulation of its fibrillation behavior and potentially improving stability.^468^ Optimization of enfuvirtide led to the discovery of the second generation agent T1249.^469^ Unfortunately, T1249 suffered from formulation challenges which led to further refinement and the discovery of sifuvirtide.^470^ Sifuvirtide is a third generation, electrostatically-constrained, α-helical peptide with potent anti-HIV-1 activity, a good safety profile, and favorable pharmacokinetic properties.^463^ Subsequent studies revealed that the emergence of resistance to sifuvirtide could be overcome by introducing methionine and threonine (MT) amino acids in the pocket-binding domain to yield MT-sifuvirtide. MT-Sifuvirtide (MTSFT) exhibited increased helicity, a higher melting temperature and an improved resistance profile when compared to its progenitor sifuvirtide. However, the binding stability and functional role of the MT-hook against wild-type and mutant forms of the HIV-1 gp41 fusion protein are unknown.^471,472^

The development of sifuvirtide offers valuable insights for future HIV-1 peptide therapeutics and PPI modulators in general. Firstly, the success of sifuvirtide highlights the importance of overcoming the challenge of peptide aggregation (fibrillation). Strategies like incorporating stabilizing elements such as the engineered salt bridges incorporated into sifuvirtide can be crucial for improving peptide stability and potentially increasing therapeutic efficacy. Secondly, sifuvirtide’s favorable pharmacokinetic profile underscores the importance of considering factors like drug absorption, distribution, metabolism, and excretion during the initial phases of peptide design. Finally, the development of sifuvirtide emphasizes the need for a balance between potency and manufacturability. While T1249 demonstrated excellent antiviral activity, formulation challenges hindered its progress. The success of sifuvirtide suggests that careful optimization of both biological and physicochemical properties is key for translating promising peptide candidates into clinically viable therapeutics. These lessons learned from sifuvirtide can be applied not only to future HIV peptide development but also to the design of PPI modulators targeting a wide range of diseases.

## Conclusion and perspective

Once considered “undruggable” due to their complexity, PPIs are now established as druggable targets in drug discovery. This paradigm shift provides a unique opportunity to develop novel therapies for a diverse range of conditions. Additionally, PPI modulators offer a promising approach to overcoming drug resistance associated with traditional therapeutics. By targeting the interactions between proteins that drive disease progression, PPI modulators can disrupt critical pathways that contribute to drug resistance. This unique mode of action does not only extend the effectiveness of current treatments but also offers new avenues for combination therapies with reduce potential for emergence of treatment resistance. However, the transition from promising targets to effective drugs is a lengthy and complex process. Initial PPI-binding fragments may lack the necessary selectivity for safe and efficacious intervention which presents a significant hurdle in the PPIs drug development.

The choice of therapeutic approach for targeting PPIs hinges on several key factors. One such factor is the characteristics of the PPI itself which plays a crucial role. For example, a PPI where there are well-defined binding sites with clear binding cavities would be an ideal target for the development of small molecule-based modulators. Conversely, PPIs where the binding interface is large and flexible necessitate bulkier modulators like peptidomimetics, peptides, or proteins for effective interaction.

The intended therapeutic target also influences the choice of therapeutic approach. Protein and peptide therapeutics face a significant limitation in crossing the blood-brain barrier (BBB) which renders them unsuitable for central nervous system (CNS) disorders. In addition to the aforementioned factors, both the desired route of administration and the therapeutic development budget are crucial considerations. Small molecule modulators are generally better suited for oral administration due to their favorable absorption properties, and they are often the more cost-effective option for drug development.

Despite the significant progress in developing PPI modulators, significant challenges remain when developing PPIs modulators. One such limitation is achieving high selectivity while minimizing off-target effects in therapeutic targets where the interaction interfaces are large and flat. The nature of these interfaces makes it difficult to design small molecules that selectively bind to a specific target without interfering with others. As such, other therapeutic options such as peptides, mAbs and proteins would be an alternate solution for targeting such targets. Moreover, optimizing the pharmacokinetic properties of PPI modulators, such as bioavailability and half-life, remains a challenging obstacle causing the failure of many therapeutic candidates.

As demonstrated by the various successful approaches described in this work, a successful strategy for developing PPI modulators in a timely fashion requires the integration of a range of techniques, including rational drug design, insights from co-crystal structures and computational techniques. Moreover, in the optimization process, seemingly minor structural changes can profoundly impact a therapeutics’ druggability and activity. The FDA approval of small molecule PPI inhibitors such as tocilizumab, siltuximab, sarilumab, satralizumab, sotorasib, adagrasib, maraviroc, venetoclax^14,50^ and fostemsavir serve as tangible evidence of the therapeutic potential of targeting PPIs. These achievements underscore the transformative impact of this approach, elevating PPIs from the realm of “potential” to the exciting reality of clinically validated targets.

Looking ahead, the rapid advancements in artificial intelligence and machine learning will significantly accelerate PPI modulator drug discovery pipeline while deepening our comprehension of PPIs as exemplified by the early results of AlphaFold 3.^473^ Combination therapies integrating PPI modulators with established treatment modalities hold promise for augmenting therapeutic outcomes and overcoming resistance. While challenges such as selectivity and off-target effects persist, ongoing innovation is addressing these hurdles. As the success rate for PPI modulators increase, this field is transitioning from a risky endeavor to a prime target for innovative therapeutics, holding significant promise for diverse disease areas.

“Welcome to the age of PPIs”.
